# Polyphenolic Profiling, Quantitative Assessment and Biological Activities of Tunisian Native *Mentha rotundifolia* (L.) Huds.

**DOI:** 10.3390/molecules24132351

**Published:** 2019-06-26

**Authors:** Imen Ben Haj Yahia, Yosr Zaouali, Maria Letizia Ciavatta, Alessia Ligresti, Rym Jaouadi, Mohamed Boussaid, Adele Cutignano

**Affiliations:** 1 Department of Biology, National Institute of Applied Science and Technology, B.P. 676, 1080 Tunis Cedex, Tunisia; 2 Institute of Biomolecular Chemistry (ICB), National Research Council (CNR), 80078 Pozzuoli (NA), Italy

**Keywords:** *Mentha rotundifolia*, *Lamiaceae*, UHPLC-MS, polyphenolics, salvianolic acid W, antioxidant activity, anti-acetylcholinesterase activity

## Abstract

Phenolic profiling of ten plant samples of *Mentha rotundifolia* (L.) Huds. collected from different bioclimatic areas of Tunisia, was for the first time carried out by using a fast ultra-high-performance liquid chromatography (UHPLC)-high resolution tandem mass spectrometry (HRMS/MS) method on a Q Exactive platform equipped with an electrospray ionization (ESI) source. An intraspecific, interpopulation variability was evidenced and a total of 17 polyphenolic metabolites were identified and quantified by using the UHPLC-HRESIMS/MS method, here validated for specificity, linearity, limit of detection, limit of quantitation, accuracy and precision. The quantitative method resulted sensitive at the nM level and reliable for rapid polyphenol quantification in vegetal matrices. The metabolomic study allowed us to identify a new compound, named salvianolic acid W, which was isolated and characterized mainly by NMR and MS analysis. A statistical correlation of the phenolic composition with antioxidant and anti-acetylcholinesterase activities was provided.

## 1. Introduction 

The genus *Mentha*, encompassing about 40 among species and recognized hybrids distributed worldwide is one of the most important genera of the *Lamiaceae* family [1]. *Mentha* species are a well-known source of terpene-rich essential oils used in traditional medicine as well as in flavoring, beverage, culinary and for cosmetic applications [2,3]. Furthermore, like other members of the *Lamiaceae* family, mint extracts contain a wealth of compounds, collectively named polyphenols, which include phenolic acids, flavones and flavanols, in a free form or as glycoconjugates, mainly responsible of the antioxidant properties of the plant. The interest towards natural polyphenolic compounds is increasing during the past years also in view of a possible technological use in the food industry as a safer alternative to synthetic molecules such as butylated hydroxyanisole (BHA) and butylated hydroxytoluene (BHT) [4]. The antioxidant potential of plant extracts and pure compounds is still the major factor in characterizing plants and, in general, nutritional health food by virtue of their bioactive components [5]. However, along with antioxidant properties, polyphenols exhibit other diverse biological activities, such as anti-inflammatory [6], anticancer [7,8], anti-atherosclerotic [9] as well as they contribute to maintaining the balance of gut microbiota [10,11]. Interestingly, *Mentha* extracts showed anti-acetylcholinesterase activity and the reversible inhibition of AChE activity has been proposed for the treatment of various diseases, including gastrointestinal disorders and Alzheimer’s disease [12,13]. In Tunisia, mint is represented by few species and namely *Mentha rotundifolia* L., *M. longifolia* (L.) Huds., *M. spicata* (*M. viridis*) L., *M. aquatica* L. and *M. pulegium* L. *M. rotundifolia* (L.) Huds. is a hybrid between *M. longifolia* (L.) and *M. suaveolens* Ehrh and has been considered as a synonym of *M. suaveolens* Ehrh [14]. Chemical studies on this species are scarce and mainly directed toward essential oils composition and bioactivity [15]. Furthermore, previous studies have demonstrated that medicinal plants growing wild in diverse environments may show differences in chemical constituents [16].

As far as we know there are no previous studies on the intraspecific, interpopulation chemical variability of Tunisian *M. rotundifolia* methanolic extracts, so the aim of the present study was to:

(i) Identify by both mass-spectrometric and NMR approaches polyphenolics from ten plant populations of Tunisian *M. rotundifolia* (L.) Huds growing wild in two bioclimatic zones;
(ii) Quantify metanolic extracts by a validated analytical strategy;
(iii) Evaluate the antioxidant and anti-acetylcholinesterase activities of extracts towards their chemical composition.

## 2. Results and Discussion

Despite that the genus *Mentha* has been largely studied for both essential oil and phenolic content, chemical characterization of polyphenols from *M. rotundifolia* has been documented in very few reports but never from Tunisian populations [17,18]. In view of exploring the chemistry and the bioactivity potential of *M. rotundifolia* from Tunisia, we carried out a collection of plant samples from different sites. The list of the *Mentha* populations labeled as MROT-1 to 10 used for the present study is reported in Supporting Material (Table S1). Extraction yields (mg extract/g dried material) ranged from 8.12% (MROT-3) to 22.54% (MROT-9; Table 1). The amount of total phenolic content (TPC) of plant extracts varied significantly (*p* < 0.05) from 5.70 (MROT-3) to 57.11 mg GAE /g DM (MROT-1). Total flavonoids (TFC) ranged from 5.12 mg ER/g DM to 24.11 mg ER/g DM (Table 1). On average, these results are in line with those reported for the same species from Algeria [19,20]. Prompted by these results showing differences in phenolic amounts among extracts, we decided to investigate their phenolic composition to get deeper insight into specific components of the polyphenolic mixture.

**Figure 1:**
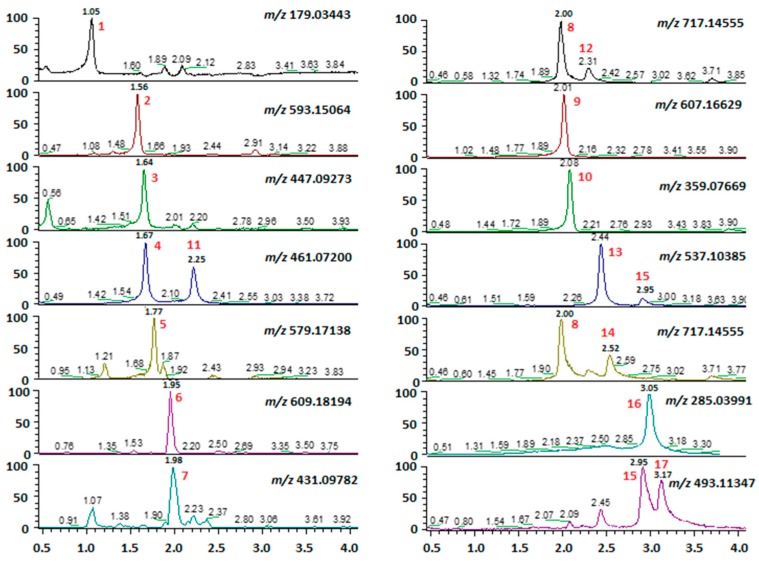
Extracted ion chromatograms (XICs) from UHPLC-HRESI-MS traces of polyphenols identified in the different populations of *M. rotundifolia*.

A small aliquot of the raw extract prepared from each population was used for a preliminary untargeted LC–MS screening. A novel ultra-high-performance liquid chromatography (UHPLC)- high resolution electrospray ionization tandem mass spectrometry (HRESIMS/MS) method was developed for the determination of the phenolic composition of the plant material. The chromatographic separation relies on a column based on Core-Shell technology (Kinetex) packed with C18-phase particles of 2.6 µm, assuring high performance, comparable to sub-2 µm particles of an UHPLC column, with significantly lower back pressure. The resolution among the various compounds detected in the extracts was optimized by using a H_2_O 0.1% FA/ACN 0.1% FA gradient. The total run time of 13 min (including 3 min for re-equilibration) was far shorter than common HPLC methods adopted in the literature for polyphenol analysis, and assured a complete elution of less polar components to avoid interference in the successive runs. The LC method was improved by the coupling with a high resolution hybrid Quadrupole-Orbitrap (Q Exactive) mass spectrometer. The mass spectrometry (MS) method workflow comprised a full MS scan followed by a set of data dependent scans with a fragmentation energy applied to gather untargeted tandem mass data along with accurate mass measurements in a single analytical run. ESI source was operating in negative ion mode and an acquisition range of 100–800 *m/z* was selected. A careful inspection of LC–MS data for each extract revealed distinct fingerprints. Furthermore, differences were evident in the relative amount of polyphenols, some of which were detectable as isomeric species (Figure 1).

**Figure 2:**
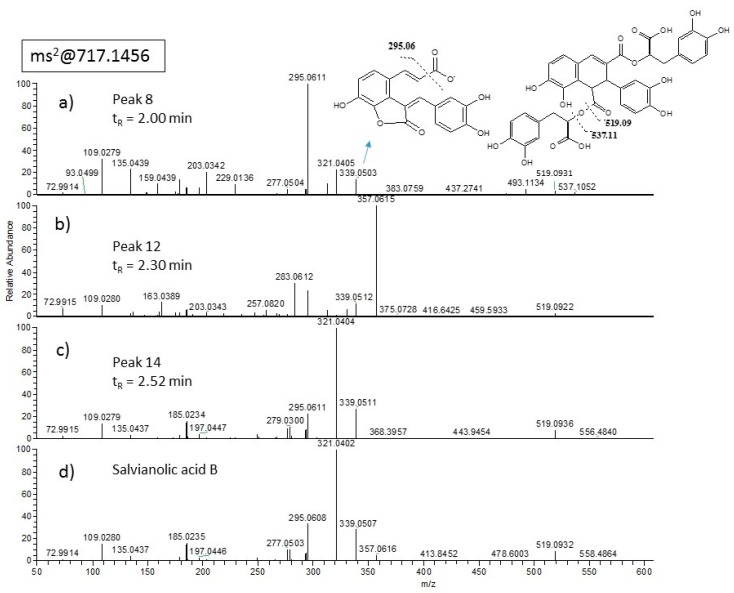
ESI-MS/MS spectra of isomeric compounds at *m/z* 717. (**a**) Peak 8, at t_R_ = 2.00 min; (**b**) peak 12 at t_R_ = 2.30 min; (**c**) peak 14 at t_R_ = 2.52 min in comparison with (**d**) salvianolic acid B pure standard eluting at t_R_ = 2.52 min.

Merging literature and experimental data, a few phenolic metabolites were tentatively listed and a pool of standard reference compounds was subsequently run to confirm molecular species identification by matching experimental data. This initial screening allowed to detect the regular presence of rosmarinic acid (peak 10), which indeed is considered as a biomarker for this genus; other polyphenolic metabolites included caffeic acid (peak 1), luteolin-7-rutinoside (peak 2), luteolin-7-glucoside (peak 3), hesperidin (peak 6), apigenin-7-glucoside (peak 7) diosmin (peak 9) and luteolin (peak 16). Isomeric species were detected in all populations analyzed, and specifically two isomers of luteolin-glucuronide at [M − H]^−^ *m/z* 461.07200, three isomers of salvianolic acid B at [M − H]^−^ *m/z* 717.14555, and two isomeric compounds at [M − H]^−^ *m/z* 537.10385 (Figure 1). By co-processing with commercially available standards, luteolin-7-glucuronide (peak 4) was identified as the peak eluting at t_R_ =1.67 min whereas its isomer, exhibiting the same fragment at *m/z* 285 and eluting at 2.25 min in our LC–MS condition, was purified by subsequent chromatographic steps (LH-20 and RP18 HPLC) and easily identified as luteolin-3′-glucuronide (peak 11) by NMR data interpretation (Figures S1–S5, Supporting Information). Three species, i.e., peaks 8, 12 and 14 eluting at t_R_ = 2.00, 2.30 and 2.52 min, respectively, exhibited [M − H]^−^ at *m/z* 717.14716 (Figure 1). Peak 8 eluting at t_R_ = 2.00 min was isolated by HPLC and identified as salvianolic acid L by interpretation of both NMR data (Figures S6–S10, Supporting Information) and MS/MS spectra (Figure 2a). The isomer eluting at t_R_ = 2.30 min (peak 12) was a minor component of the extracts, not isolated during this work and here generally indicated as isosalvianolic acid B (Figure 2b). The peak 14 eluting later (Figure 2c) was identified by both retention time and MS/MS analysis as salvianolic acid B by comparison with commercial standard (Figure 2d). 

**Figure 3:**
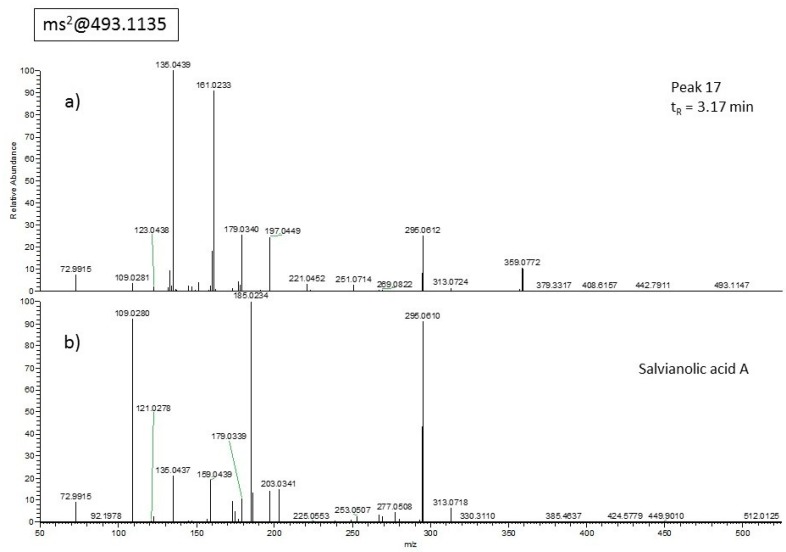
ESI-MS/MS spectra of compound at *m/z* 493. (**a**) Peak 17 at t_R_ = 3.17 min compared with (**b**) salvianolic acid A pure standard eluting at t_R_ = 2.86 min.

A compound with molecular ion at *m/z* 493.11462 eluting at t_R_ = 3.17 min (Figure 3a, peak 17) did not correspond to the reference compound salvianolic acid A (Figure 3b), which indeed eluted at t_R_ = 2.86 min. Therefore, it was labeled as isosalvianolic acid A. Furthermore, a metabolite at *m/z* 579.17224 originally identified in our analysis as naringin resulted to be its structural isomer, exhibiting different retention time but identical fragmentation when compared to naringin standard and therefore was indicated as isonaringin (peak 5, t_R_ = 1.77 min). 

**Figure 4:**
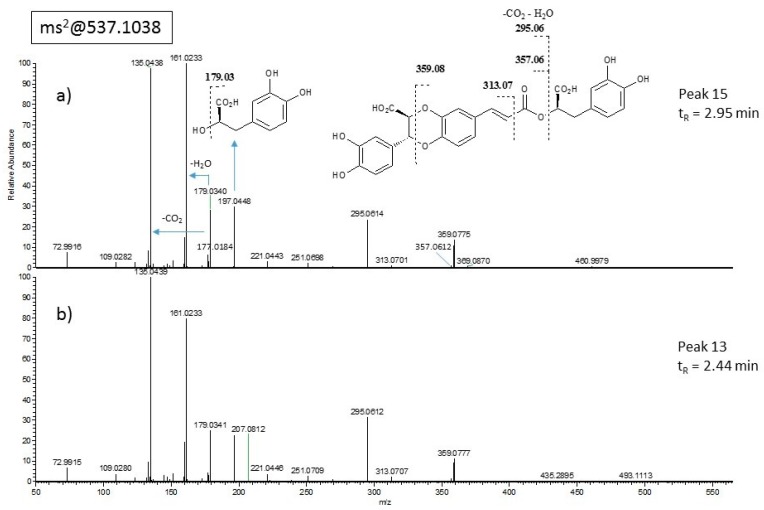
ESI-MS/MS spectra of compound at *m/z* 537 exhibiting the same fragmentation pattern. (**a**) Peak 15 at t_R_ = 2.95 min and (**b**) peak 13 at t_R_ = 2.44 min.

**Figure 5:**
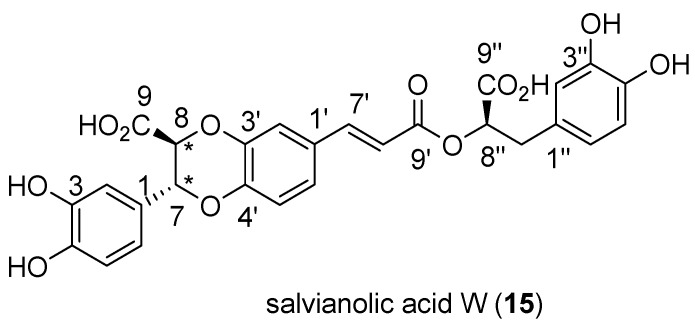
Structure of novel salvianolic acid W (**15**) detected in all Tunisian *M. rotundifolia* populations analyzed.

Finally, two peaks eluted at t_R_ = 2.44 (peak 13) and 2.95 min (peak 15) exhibiting the same molecular ion at *m/z* 537.10352 and fragmentation pattern (Figure 4). The compound eluting at t_R_ = 2.95 min (Figure 4a) was isolated by HPLC and fully characterized by a combination of spectroscopic and spectrometric methods, resulting in a new diastereoisomer of salvianolic acid J, here named salvianolic acid W (**15**, Figure 5) and chemically described below. The isomeric compound **13** degraded during HPLC isolation; therefore it remains uncharacterized and is here indicated as isosalvianolic acid W. 

Compound **15** (Figure 5) was isolated as an optically active yellowish amorphous solid with [α]_D_ 10.0 (c = 0.1, MeOH), displaying UV absorption at 209, 219, 289 and 324 nm. Its molecular ion [M − H]^−^ measured at *m/z* 537.1038 by HR-ESIMS indicated the molecular formula C_27_H_22_O_12,_ and was accompanied even in mild ionization conditions by an in-source fragment ion at *m/z* 493.1152 due to the loss of CO_2_, suggesting the presence of a COOH group in the structure. ^1^H and ^1^H,^1^H-COSY NMR data (600 MHz, CD_3_OD; Table 2) revealed three sets of ABX coupled systems, i.e., δ 6.88 (1H, d, 1.8, H-2), 6.75 (1H, d, 8.1, H-5) and 6.79 (1H, dd overlapped, H-6); 7.16 (1H, d, 1.8, H-2’), 6.96 (1H, d, 8.4, H-5’) and 7.12 (1H, dd, 8.4, 1.8, H-6’); 6.80 (1H, d, overlapped, H-2″), 6.70 (1H, d, 8.1, H-5″) and 6.66 (1H, dd, 8.1, 1.8, H-6″), which were attributed by combining HSQC and HMBC data to three catechol residues. In the olefinic region was present a *trans* coupling proton system at δ 7.57 (1H, d, 16.0, H-7’) and 6.38 (1H, d, 16.0, H-8’) conjugated to a carboxyl functionality at 168.8 ppm (C-9’), which indicated the occurrence of a caffeoyl-unit. On the other hand, an oxygenated α-carboxyl methine proton appeared at δ 5.11 (dd, 10.0, 3.2, H-8″) coupled to the methylene protons at δ 2.95 (dd, 10.0, 14.3, H-7″a) and 3.12 (dd, 3.2, 14.3, H-7″b). HMBC correlations with a carboxyl group at 176.9 ppm (C-9″) and the aromatic carbons at 131.3 (C-1″), 117.1 (C-2″) and 121.7 (C-6″) ppm, supported the presence of a (3,4-dihydroxyphenyl)-lactic moiety. Completed the proton resonances an isolated spin system constituted by two oxymethine at δ 5.16 (1H, d, 5.5, H-7) and 4.52 (1H, d, 5.5, H-8) attributed to a 7,8-disubstituted benzodioxane subunit. Diagnostic HMBC correlations between H-7 and the aromatic carbons 129.9 (C-1), 115.6 (C-2) and 120.2 ppm (C-6) and between H-8 and the quaternary carbons at 174.5 (C-9) and 146.8 ppm (C-3’) allowed to place the phenyl ring and the carboxyl function on carbons C-7 and C-8, respectively. The planar structure resembled the one described for salvianolic acid J [21]. In particular, the junction of the benzene and the dioxane rings was secured by diagnostic hetero-correlations observed for H-7 and H-6’ with C-4’ (145.8 ppm) thus excluding the other possible junctional-isomer reported as salvianolic acid P [22]. However, the coupling constant of 5.5 Hz between H-7 and H-8 suggested a *trans*-orientation instead of *cis* as in salvianolic acid J for these two protons on the dioxane cycle [23,24].

The pattern of fragmentation of molecular ion observed in ESIMS/MS spectra (Figure 4a) was in agreement with the structure depicted. In fact, diagnostic fragments at *m/z* 359.0775 and 357.0612 were compatible with the breakage of the dioxane ring and of the ester bond, generating the (dehydro)-caffeoyl acid fragments at *m/z* 177.0184 and 179.0340, respectively. Absolute stereochemistry of carbon C-8″ was assumed as *R* in accordance with rosmarinic acid stereochemistry reported so far. Hence, compound **15** resulted in a novel diastereoisomer of salvianolic acid J, here named salvianolic acid W (Figure 5).

**Figure 6:**
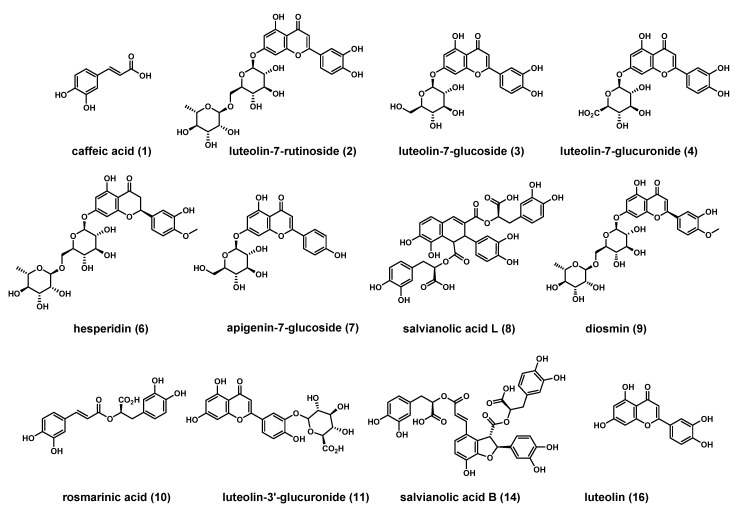
Structures of known compounds identified in *M. rotundifolia*.

On the whole, 17 polyphenolics were identified (Figure 6) and among these eight were recurrent in all the examined extracts, i.e., the phenolic acids caffeic acid (**1**), salvianolic acid L (**8**) rosmarinic acid (**10**), isosalvianolic acid A (17) and novel salvianolic acid W (**15**) together with flavonoids represented by luteolin-7-glucuronide (**4**), luteolin-3’-glucuronide (**11**) and luteolin (**16**). Furthermore, luteolin-7-rutinoside (**2**), luteolin-7-glucoside (**3**), isonaringin (**5**), hesperidin (**6**), apigenin-7-glucoside (**7**) and salvianolic acid B (**14**) were for the first time reported in *M. rotundifolia*.

To quantify the polyphenols, we set up a quantitative method by using a pool of commercially available reference standards. Sinapic acid, which resulted absent in all natural samples analyzed in the untargeted qualitative pre-screening, was chosen as internal standard (IS). The LC–MS method was validated according to ICH Q2 (R1) guidelines. It is suitable for quantitative measurement of polyphenolic compounds in *Mentha* and in other similar matrices. Validation parameters were reported in Table 3. The linearity of the method was evaluated by analyzing for each standard seven calibration points in triplicate over the nominal range. All standard curves showed good linearity with R^2^ values within 0.9966–0.9995. Limit of detection (LOD) and limit of quantitation (LOQ) varied greatly among the different molecular species, ranging from 4.7/14.4 ng/mL, respectively, for diosmin to 75.5/229.0 ng/mL, respectively, for salvianolic acid B. Recovery was established on sinapic acid and was 62.4% ± 20.2%. The precision, expressed as the relative standard deviation (RSD; %), met the acceptance criteria, being always below 15% for all calibration points in both intra- and inter-assay measurements. Accuracy values were all within 100% ± 15% range. Quantitative measurement of polyphenolic levels was achieved by internal standard approach and results are reported in Table 4. Differences in occurrence and concentration (mg/g extract) of the identified compounds among the ten populations were observed. However, polyphenolic acids represented by caffeic acid (**1**), rosmarinic acid (**10**), salvianolic acid L (**8**), salvianolic acid W (**15**), isosalvianolic acid A (**17**) and flavonoids represented by luteolin-7-glucuronide (**4**), luteolin-3’-glucuronide (**11**) and luteolin (**16**), were always detected in all samples. Therefore, these components could be considered as polyphenolic biomarkers for this Tunisian species. Other polyphenolics occurred at different concentrations in various samples, some of them are almost ubiquitous (e.g., luteolin-7-rutinoside (**2**) and luteolin-7-glucoside (**3**)) others are scattered (e.g., salvianolic acid B (**14**), hesperidin (**6**), isonaringin (**5**) and diosmin (**9**)).

The population MROT-1 and -2 from the two localities Tamra and Oued maaden exhibited the highest contents of polyphenols, with major compounds represented by rosmarinic acid (**10**), salvianolic acid L (**8**), luteolin-7-glucuronide (**4**) and luteolin-3’-glucuronide (**11**). Isosalvianolic acid B (**12**) and isosalvianolic acid W (**13**) were also abundant and at the highest concentration in MROT-1 compared to the other populations. MROT-7 exhibited the highest amount of caffeic acid (**1**) per g of dry extract (1.38 mg/g) and MROT-3 exhibited the uppermost content in luteolin (**16**; 4.91 mg/g) and diosmin (**9**; 3.95 mg/g). The highest amount of hesperidin (**6**; 10.0 mg/g) was detected in MROT-4.

The extraction yield and chemical variability evidenced in the polyphenolic composition of populations of *M. rotundifolia* may be traced back to the influence of various climatic and edaphic conditions on the biosynthetic pathways responsible for the production of compounds related to the adaptive strategy of plants against environmental constraints [25]. Changes in environmental conditions may occur also over short distances thus supporting the differences we observed among populations even from the same bioclimatic zone (lower humid or upper semi-arid).

Phenolic acids and flavonoids are associated with potent antioxidant activity as well as with a plethora of beneficial effects on the human health. We evaluated the antioxidant activity of Tunisian mint extracts in vitro by three different tests, namely free radical scavenging assay (DPPH assay), β-carotene bleaching inhibition and ferric ion reducing antioxidant power (FRAP). Results of antioxidant assays are reported in Table 1. Antiradical (DPPH) activity expressed as IC50 (µg/mL) varied significantly among the populations tested, from 15.16 (MROT-1) to 96.66 µg/mL (MROT-8). Three populations showed antioxidant potential (MROT-1, 4 and -7) of 15.16, 21.40 and 40.66 µg/mL, respectively, which resulted significantly in comparison with the standard (Trolox = 3.77 μg/mL). Previous studies on the same species reported similar DPPH potential [17,19,26]. As for the β-carotene bleaching assay, again MROT-1 along with MROT-2 and -6 showed the highest inhibition with an IC50 of 106.66, 115.08 and 110.93 µg/mL, respectively. Literature data on *M. rotundifolia* extracts in this test are quite discordant, varying from very low to extremely high activity [17,27]. However, data on *M. rotundifolia* are often difficult to compare due to differences in adopted methodologies [28]. The FRAP values also ranged remarkably from 62.02 µmol Fe^2+^/g in MROT-3 to 574.03 µmol Fe^2+^/g in MROT-1. This latter population definitely resulted the most promising for antioxidant potential.

Polyphenols have also emerged as possible candidates for the treatment of gastrointestinal and neurodegenerative disease by virtue of their reversible inhibitory effects on AChE [29,30]. Few *Mentha* species have been tested for the inhibition of AChE activity, i.e., *M. spicata, M. pulegium* and *M. piperita* showing an IC50 in the range 0.72–1.93 mg/mL [12]. This activity has been associated to rosmarinic acid or other phenolic acids [12,31,32] or to flavonoids, which exhibited similar or even more potent activity [18,30,33]. The ten *M. rotundifolia* extracts exhibited moderate anticholinesterase activities in comparison with the positive control (Donepezil: 18.0 ± 0.1 µg/mL) (Table 1). The highest values attributed to MROT-2, -3, -4 and -5 were around 0.2 mg/mL, which were higher than those reported in the literature for other *Mentha* species. 

The correlation analysis (Table 5) showed that phenolic acids represented by caffeic acid (r = –0.48, *p* < 0.05), rosmarinic acid (r = –0.47, *p* < 0.05), isosalvianolic acid B (r = –0.41, *p* < 0.05), salvianolic acid L (r = –0.56, *p* < 0.01), salvianolic acid W (r = –0.58, *p* < 0.01), isosalvianolic acid A (r = –0.61, *p* < 0.01) and isosalvianolic acid W (r = –0.51; *p* < 0.01) and flavonoid pool constituted by luteolin (r = –0.37, *p* < 0.05), luteolin-7-glucoside (r = –0.45, *p* < 0.01), luteolin-7-glucuronide (r = –0.51, *p* < 0.01), luteolin-3’-glucuronide (r = –0.45, *p* < 0.01), apigenin-7-glucoside (r = –0.40, *p* < 0.05) and isonaringin (r = –0.65, *p* < 0.01) were negatively correlated to DPPH. 

A positive correlation was found between isosalvianolic acid B (r = 0.31, *p* < 0.05), salvianolic acid L (r = 0.30, *p* < 0.01), salvianolic acid W (r = 0.34, *p* < 0.01), isosalvianolic acid A (r = 0.41, *p* < 0.05), isosalvianolic acid W (r = 0.41, *p* < 0.05) and FRAP. Rosmarinic acid (r = −0.49, *p* < 0.05), isosalvianolic acid B (r = −0.51, *p* < 0.01), salvianolic acid L (r = −0.49, *p* < 0.05), salvianolic acid W (r = −0.31, *p* < 0.05), isosalvianolic acid W (r = −0.30, *p* < 0.05), luteolin-7-glucoside (r = −0.39, *p* < 0.05), luteolin-7-glucuronide (r = −0.61, *p* < 0.01), luteolin-3’-glucuronide (r = −0.65, *p* < 0.01) and apigenin-7-glucoside (r = −0.58, *p* < 0.05) were also found to be negatively correlated to the β-carotene bleaching activity. The population MROT-1, exhibiting the highest amounts in phenolic acids and in the majority of flavonoids, showed the best antiradical, β-carotene bleaching and ferric reducing capacities. Based on the above statistical analysis, this higher antioxidant activity seems to be related to its richness in phenolic compounds and not to a specific composition.

Differently from antioxidant activity, anti-cholinesterase activity appeared associated to specific compounds. Salvianolic acid B (r = −0.30, *p* < 0.05), hesperidin (r = −0.48, *p* < 0.05), luteolin (r = −0.65, *p* < 0.01) and diosmin (r = −0.54, *p* < 0.1) were negatively correlated to the anticholinesterase activity (Table 5). The populations MROT-2, -3, -4 and MROT-5 exhibiting the highest anti-AChE activities, were the richest in at least one of these compounds. Katalinic et al. reported that a high AChE inhibition potency is attributed to luteolin [34]. Hesperidin was previously found to exhibit cholinesterase inhibition [35] while pure diosmin was proposed as agent for memory restoration, in treatment of dementia [36]. A correlation was also specifically observed with salvianolic acid B, whose role in AChE inhibition and possible development in drugs against neurological diseases has been recently documented [32].

The compounds putatively responsible of the anti-acetylcholinesterase activity observed in the extracts are not the most abundant in these populations. This result, in agreement with the general finding that flavonoids are AChE inhibitors, highlights that specific components, although minor, could be responsible of the observed bioactivity.

## 3. Material and Methods

### 3.1. General

Optical rotations were measured on a Jasco P2000 digital polarimeter. UV spectra were acquired on a Jasco V-650 Spectrophotometer, CD spectra were registered on a Jasco J-815 polarimeter. NMR spectra were recorded on a Bruker Avance DRX 600 operating at 600 MHz for proton, equipped with an inverse TCI CryoProbe fitted with a gradient along the Z-axis or on an Avance III HD operating at 400 MHz for proton, equipped with a CryoProbe Prodigy. Chemical shifts values are reported in ppm (δ) and referenced to internal signals of residual protons (for CD_3_OD ^1^H δ 3.34, ^13^C 49.0 ppm). HPLC separations were performed on a Shimadzu high-performance liquid chromatography system using a Shimadzu liquid chromatograph (Shimadzu, Kyoto, Japan) LC-20ADXR equipped with a Diode Array Detector SPDM-20A and a Kromasil RP-18 column 250 mm × 10 mm, 5 µm (Phenomenex, Castel Maggiore (BO), Italy). Polyphenolic standards (purity > 98%), formic acid (LC–MS grade) Folin-Ciocalteu reagent, sodium carbonate (Na_2_CO_3_), aluminum chloride (AlCl_3_), 2,2-diphenyl-1-picrylhydrazyl, 2,4,6-*tris*-(2-pyridyl)-*S*-triazine (TPTZ), lyophilized acetylcholinesterase (AChE, electric eel, type VI-S) and acetylthiocholine iodide (ATCI) were purchased from Sigma Aldrich. ACN LC–MS grade was purchased from Merck. *β*-carotene was obtained from Fluka. 5,5’-dithio-bis- [2-nitrobenzoic acid] (DTNB) was purchased from MP Biomedicals. Water for LC–MS was obtained by a MilliQ apparatus (Millipore, Milan, Italy).

### 3.2. Plant Collection and Extraction

Aerial parts of plants taxonomically identified as *M. rotundifolia* (L.) Huds. were collected in February 2014 from diverse geographical regions of Tunisia (Table S1). The samples were collected from rivers, lands and temporarily flooded areas. Ten individuals per population were harvested. Ten populations (MROT-1 to MROT-10) were considered. Voucher specimens (M.r.N°1-10, INSAT14) were deposited at the Herbarium of the Laboratory of National Institute of Applied Sciences (Tunis, Tunisia). The plants were dried at room temperature for two weeks. Methanolic extracts for chemical measurements and biological assays were prepared using 1 g of dry leaves. After maceration in 10 mL of methanol for 24 h at room temperature, the samples were filtered, dried under vacuum and the extracts stored at −20 °C until analysis.

### 3.3. Total Phenolic Content (TPC) and Total Flavonoid Content (TFC).

The total phenols for each individual were determined using a spectrophotometric method [37]. An aliquot of each diluted sample extract (0.5 mL) was mixed with 2 mL Folin-Ciocalteu reagent. After 5 min, 2.5 mL of sodium carbonate solution (7.5%) was added. After incubation (90 min) in dark, the absorbance of samples versus that of the blank was read at 760 nm. Total phenols were expressed as gallic acid equivalents (mg GAE/g DW).

The total flavonoid content was determined according to Chetoui et al. [38]. One mL of the sample was mixed with 1 mL of 2% AlCl_3_. After incubation for 15 min, the absorbance was measured at 430 nm. The percentage content of flavonoids was expressed as mg rutin equivalent/g DW (mg ER/g DW), using the calibration curve of rutin (0–400 µg/mL range).

### 3.4. Identification and Quantification of Phenolic Compounds

#### 3.4.1. Identification of Phenolic Compounds

For isolation and identification of metabolites, air-dried aerial parts (18 g) of *M. rotundifolia* from Tamra (MROT-1) were powdered and exhaustively extracted at room temperature with a hydro-alcoholic solution (MeOH/H_2_O, 80:20, 200 mL × six times). The extracts obtained were combined and concentrated under vacuum to afford an aqueous solution (100 mL), which was sequentially partitioned with *n*-hexane (100 mL × six times), ethyl acetate (EtOAc; 100 mL × five times) and *n*-butanol (*n*-BuOH; 60 mL × four times). The corresponding three crude extracts from *n*-hexane (0.32 g), EtOAc (0.28 g) and *n*-BuOH (0.50 g) were obtained. The butanolic extract was loaded onto a LH-20 Sephadex column (150 cm length, 3 cm diameter) packed and eluted isocratically with MeOH. Homogeneous fractions were combined to give 12 sub-fractions (A-N). Isolation of pure metabolites was carried out by HPLC on a reversed phase Kromasil RP-18 column (250 mm × 10 mm) using a gradient elution of A (water/ACN 75:25, 0.25% FA) and B (ACN, 0.25% FA) as follows: 0–3 min, 100% A; 3–11 min, 65% A: 35% B; 11–18 min 65% A: 35% B; 18–22 min 100% B; 22–28 min, 100% B. Flow 2 mL/min. PDA detection 190–800 nm, extracted wavelength at 280 nm. Salvianolic acid W (**15**, 1.0 mg, t_R_ = 14.6 min) was purified from LH-20 fraction M (25 mg); luteolin-3’-glucuronide (**11**, 1.1 mg, t_R_ = 12.2 min) and salvianolic acid L (**8**, 2.5 mg, t_R_ = 11.8 min) were obtained from LH-20 fraction N (10 mg).

Salvianolic acid W (**15**). NMR data: see Table 2. HR-ESIMS [M − H]^−^ 537.10386 *m/z*, C_27_H_22_O_12_ (calc. 537.10385); [α]_D_ 10.0 (c = 0.1, MeOH); UV (MeOH) λ_max_ (ε): 219 (8740), 289 (5795), 324 (5560) nm; CD (c = 0.125 mg/mL) MeOH λ_max_ (θ): 222 (11400), 240 (−1562), 255 (285), 276 (−1591), 296 (5492), 330 (−898) and 351 (516) nm. 

Identity of compounds in raw extracts was based on comparison of retention time, high resolution *m/z* measurements and fragmentation pattern with pure standards, when available. Identification of isomeric compounds was performed on the basis of HR-ESIMS and fragmentation data. Identification of salvianolic acid L, luteolin-3′-glucuronide and the new salvianolic acid W, were based on spectroscopic and spectrometric data of pure compounds obtained by HPLC.

#### 3.4.2. Quantification of Phenolic Compounds

For the quantification of polyphenols, *Mentha* samples (0.05 g dried leaves) spiked with 125 µg of IS were extracted with methanol (3 mL × 1 mL). The extracts were dried under vacuum, resuspended in 1 mL of MeOH and diluted 1:25 for LCMS analysis to obtain a final IS concentration of 5 µg/mL. MS recovery was established on IS by comparing the response of a defined amount (5 µg/mL) in *Mentha* samples spiked before and after extraction. Structural isomers were quantified by using the calibration curve of the corresponding known standard compounds. UHPLC analysis was performed on Infinity 1290 UHPLC System (Agilent, Milan, Italy). Chromatographic separation was achieved on a Kinetex Core-Shell C18 column (75 mm × 2.1 mm, 100 A, 2.6 µm) (Phenomenex, Castel Maggiore (BO), Italy). Elution solvents: (A) water 0.1% FA, (B) ACN 0.1% FA. Gradient: 0–1 min, 10% to 20% B; 1–8 min to 50% B; 8–8.5 min to 100% B; 8.5–10 min 100% B; then in 1 min return to initial condition and equilibration for 2 min. Flow 0.6 mL/min. The injection volume was 5µL. The UHPLC system was coupled to Q Exactive Mass Spectrometer (Thermo Scientific, San Jose, CA, USA) equipped with a HESI source operating in negative ion mode. Spectra were acquired over the range 100–800 *m/z*. Optimum values were as follow: Spray voltage 3 kV; Capillary temperature 320 °C; S-lens RF level 60; Aux gas heater temp 320 °C; Sheath gas flow rate 50; Aux gas flow rate 30. Resolution in Full Scan 70000, MS/MS experiments was performed with NCE at 20, 30 and 40. Resolution in MS/MS mode was set at 17500. MS data were processed by Xcalibur Software (vers. 3.0.63, Thermo Scientific, San Jose, CA, USA). 

##### Validation of the UHPLC-MS Quantitative Method

The validation of the analytical procedure was performed in accordance with ICH guidelines considering as validation characteristics: Linearity, range, detection limit (LOD), quantitation limit (LOQ), precision and accuracy. Seven calibration solutions in the range 0.125–10 µg/mL containing a pool of polyphenolic standards with a spiked amount of 5 µg/mL of IS (sinapic acid), were prepared by serial dilution of a stock solution of 2 mg/mL in MeOH. Each analysis was performed in triplicate. Results were plotted considering as response the area ratio of each polyphenol standard/IS. Peak area was measured on the extracted ion chromatogram (XIC) of molecular ion [M − H]^−^. A least-square linear regression weighting by the reciprocal of the concentration was used to best fit the linearity curve. LOD and LOQ were calculated by considering the standard deviation of the response (σ) and the slope (S) of the calibration curve: LOD was expressed as 3.3 σ/S and LOQ as 10 σ/S. Recovery was calculated as percent ± SD on sinapic acid, spiked in the organic matrix before and after methanol extraction, in duplicate for each sample. QC samples were prepared at three different concentrations for each phenolic standard (low, middle and high) of the working range and used for repeatability and intermediate precision and accuracy. Each solution was injected six times on the same day for intra-assay precision and three times for three consecutive days for intermediate precision. The instrumental precision was expressed as percentage of relative standard deviation (% RSD). Accuracy was evaluated with the above QC samples within day and inter-day as percentage of the ratio between measured mean concentration and nominal concentration.

### 3.5. Antioxidant Activity

The antioxidant activity was assessed by 1,1-diphenyl-2-picrylhydrazyl (DPPH), β-carotene bleaching method systems and ferric reducing ability (FRAP).

#### 3.5.1. Free Radical-Scavenging Assay

The free radical-scavenging activity of methanolic extracts was evaluated with the DPPH assay [39]. Three mL of 4.10^−5^ M DPPH were added to 1 mL of the extract at different concentration. The mixture was shaken and allowed to stand at room temperature for 30 min. The decrease in absorbance at 517 nm was measured against a blank. The radical-scavenging activity of samples, expressed as percentage inhibition of DPPH, was calculated according to the formula:

% inhibition = [(AB − AA)/AB] × 100(1)

where AB and AA are the absorbance values of the control and of the test sample, respectively.

#### 3.5.2. β-Carotene Bleaching Assay

The β-carotene method was carried out according to Mata et al. [12]. Two mL of β-carotene solution (0.2 mg/mL in chloroform) were pipetted into around bottomed flask containing 20 µL linoleic acid and 200 µL Tween 20. The mixture was then evaporated at 40 °C for 10 min to remove the solvent, the addition of distilled water (100 mL) followed immediately. After agitating the mixture, 1.5 mL aliquot of the resulting emulsion was transferred into test tubes containing 150 µL of extract and the absorbance was measured at 470 nm against a blank. The tubes were placed in a water bath at 50 °C and the oxidation of the emulsion was monitored by measuring absorbance at 470 nm over a 60 min. The same procedure was repeated with the synthetic antioxidant, butylhydroxytoluene (BHT) as positive control. The antioxidant activity (%) was evaluated in terms of bleaching of β-carotene using the following formula:

% Inhibition = [(AA_t120_ − AB_t120_)/(BB_t0_ − AB_t120_)] × 100 (2)

where AA and AB are the absorbance values measured for the test sample and control, respectively, after incubation for 120 min (t120), and BB is the absorbance value for the control measured at time zero (t0). A concentration of extract providing 50% inhibition (IC50) was obtained plotting inhibition percentage versus extract solution concentrations.

#### 3.5.3. Ferric Reducing Power Activity (FRAP Assay)

The ferric reducing ability was assessed following the method described by Benzie and Strain [40]. FRAP reagent containing 2.5 mL of 10 mM of 2,4,6-tris(2-pyridyl)-1,3,5-triazine (TPTZ) solution in 40 mM HCl plus 2.5 mL of 20 mM FeCl_3_ and 25 mL of 0.3 M acetate buffer (pH 3.6) was warmed prior to the analysis. FRAP Reagent (900 µL) was mixed with 90 µL distilled water and 30 µL of diluted extracts (1:10 *v*/*v*) and then was warmed to 37 °C in a water bath for 30 min, and the absorbance was read at 593 nm. A standard curve was prepared using different concentrations of FeSO_4_–7H_2_O (200–2000 µmol/L). Results were corrected for dilution and expressed in mmol Fe^2+^/g of plant extract.

### 3.6. Acetylcholinesterase Inhibition Assay

The anti-acetylcholinesterase activity was measured using an adaptation of the methods described by Eldeen et al. [41] and Ferreira et al. [42]. Briefly, 355 µL of Tris-HCl buffer (50 mM, pH 8; containing 0.1% bovine serum albumine), 20 µL of methanolic extract (at different concentrations) and 25 µL of the enzyme solution (AChE, 0.28 U/mL) were incubated during 15 min. Subsequently, 100 µL of AChI solution (0.15 mM) and 500 µL of DTNB (0.3 mM) were added. The final mixture was incubated for another 30 min at 37 °C. Absorbance of the mixture was measured at 405 nm. A control mixture was performed without addition of the extract. The anti-acetylcholinesterase activity was calculated using the following formula:

AChE inhibition (%) = [(A_c_ − A_s_)/A_c_] × 100 (3)

where, A_c_ and A_s_ are the absorbance of the control and the sample, respectively. All tests were performed in triplicate and results were expressed as IC50 (concentration providing 50% AChE inhibition) obtained by plotting the methanolic extract concentration versus inhibition percentage. Donepezil was used as positive control.

### 3.7. Statistical Analyses

Correlation analysis (CA) with PROC CORR procedure 9.3.1 (SAS, Cary, NC, USA) was used. All determinations were performed in triplicates and results were expressed as mean ± standard deviation.

## 4. Conclusions

The present study reports for the first time a comprehensive analysis of the phenolic composition in ten different Tunisian native populations of *M. rotundifolia* (L.) Huds. (MROT-1 to 10). The raw extracts, profiled and quantified by a novel and here validated UHPLC-HRESIMS analysis based on a Q Exactive platform, showed significant qualitative and quantitative phenolic variability among the analyzed populations. Few polyphenolic acids were recurrent in all the examined extracts, i.e., rosmarinic acid, caffeic acid, salvianolic acid L, isosalvianolic acid A and the novel salvianolic acid W. Common flavonoids were represented by luteolin and its glucuronides. Furthermore, for the first time salvianolic acid B, luteolin glicosides, apigenin-7-glucoside, hesperidin and isonaringin were reported from *M. rotundifolia*. This different chemical composition was correlated to the antioxidant and anticholinesterase potential of the extracts. The population MROT-1 from Tamra (Beja) displaying the highest contents in all investigated polyphenolic classes, exhibited the highest antioxidant activity of the polyphenolic extracts evaluated by the DPPH, β-carotene and FRAP tests. This population could be selected as starting material for crop improvement program exploiting antioxidant potential of mint extracts. On the other hand, the highest anticholinesterase inhibition activity observed for populations MROT-2, -3, -4 and -5 was correlated to the presence of salvianolic acid B, luteolin, hesperidin and/or diosmin.

The wide range and heterogeneity of the ecological factors characterizing the sites surveyed seem to influence the content of the compounds. The ongoing studies on the genetic diversity based on molecular markers of populations cultivated in the same ecological conditions should help in clarifying chemical variability, in selecting interesting genotypes and optimizing their use for human health.

## Figures and Tables

**Table 1:** Extraction yield, total polyphenol content, total flavonoid content, antioxidant activity (DPPH, β-carotene and ferric reducing power activity—FRAP) and AChE inhibition of extracts from *Mentha rotundifolia*.

	MROT-1	MROT-2	MROT-3	MROT-4	MROT-5	MROT-6	MROT-7	MROT-8	MROT-9	MROT-10
Extraction Yield (%)	17.79 ± 2.16 ^c^	14.83 ± 0.47 ^d^	8.12 ± 0.57 ^f^	14.84 ± 1.37 ^d^	13.37 ± 2.63 ^e^	13.92 ± 1.33 ^e^	19.26 ± 3.62 ^b^	17.15 ± 0.03 ^c^	22.54 ± 2.73 ^a^	19.54 ± 0.81 ^b^
Polyphenols (mg GAE/g DW)	57.11 ± 1.53 ^a^	39.47 ± 0.28 ^b^	5.70 ± 0.39 ^f^	30.09 ± 1.19 ^cd^	33.17 ± 3.96 ^c^	25.76 ± 0.49 ^ed^	33.30 ± 3.05 ^c^	22.61 ± 0.07 ^e^	24.80 ± 1.44 ^ed^	20.55 ± 1.35 ^e^
Flavonoids (mg ER/g DW)	24.11 ± 0.33 ^a^	21.65 ± 0.09 ^a^	5.12 ± 0.29 ^d^	23.66 ± 2.24 ^a^	23.73 ± 2.34 ^a^	16.28 ± 1.96 ^b^	20.55 ± 0.02 ^a^	10.92 ± 0.01 ^c^	20.20 ± 0.36 ^a^	11.79 ± 0.41 ^c^
DPPH (IC50 µg/mL)	15.16 ± 0.16 ^e^	57.44 ± 3.87 ^c^	83.50 ± 0.95 ^b^	21.40 ± 0.85 ^e^	54.40 ± 0.40 ^c^	62.26 ± 9.64 ^c^	40.66 ± 0.66 ^d^	96.66 ± 3.33 ^a^	75.50 ± 3.40 ^b^	86.50 ± 0.97 ^ab^
β-carotene (IC50 µg/mL)	106.66 ± 6.66 ^f^	115.08 ± 9.34 ^f^	816.66 ± 11.78 ^b^	902.50 ± 22.74 ^a^	936.25 ± 0.69 ^a^	110.93 ± 10.96 ^f^	796.66 ± 26.66 ^b^	196.66 ± 20.27 ^e^	745.55 ± 4.37 ^c^	458.33 ± 1.82 ^d^
FRAP (µmol eqFe^2+^/g)	574.03 ± 0.98 ^a^	154.54 ± 9.81 ^h^	62.02 ± 0.95 ^j^	497.43 ± 21.31 ^b^	251.15 ± 2.22 ^f^	115.06 ± 13.77 ^i^	372.11 ± 0.01 ^d^	289.65 ± 1.12 ^e^	209.94 ± 11.43 ^g^	445.41 ± 12.67 ^c^
AChE (IC50 mg/mL)	0.70 ± 0.03 ^d^	0.24 ± 0.01 ^fg^	0.21 ± 0.01 ^g^	0.27 ± 0.01 ^f^	0.21 ± 0.01 ^g^	0.83 ± 0.02 ^c^	0.87 ± 0.03 ^c^	0.93 ± 0.01 ^b^	0.54 ± 0.02 ^e^	2.16 ± 0.02 ^a^

Values were means of three replications ± SD. Extraction yield (%): (mg extract × 100)/g dry material. DPPH scavenging activity: IC50 values for the positive control Trolox: 3.77 ± 0.12 μg/mL; β-carotene bleaching inhibition: IC50 values for the positive control BHT: 29.40 ± 0.11 μg/mL; FRAP potential: µmol FeS0_4_.7H_2_0/g extract; AChE activity: IC50 values for the positive control Donepezil: 18.0 ± 0.1 µg/mL. Values in lines followed by the same letter are not significantly different (Duncan’s test, *p* < 0.05).

**Table 2:** NMR data of salvianolic acid W (**15**; 600 MHz).

C		^1^H, δ, m, *J* (Hz) ^a^	^13^C, ppm ^a^	^1^H, δ, m, *J* (Hz) ^b^	^13^C, ppm ^b^
**1**	C	-	129.9	-	129.5
**2**	CH	6.88, d, 1.8	115.6	6.98, d, 1.1	115.2
**3**	C	-	146.2	-	144.0 ^g^
**4**	C	-	146.8 ^e^	-	145.4
**5**	CH	6.75, d, 8.1	116.2	6.85 ^d^	116.7
**6**	CH	6.79 ^c^	120.2	6.84 ^d^	120.1
**7**	CH	5.16, d, 5.5	78.2	5.28, d. 4.9	77.0
**8**	CH	4.52, d, 5.5	81.1	4.69, d, 4.9	79.2
**9**	C	-	174.5	-	174.5
**1′**	C	-	129.3	-	128.6
**2′**	CH	7.16, d, 1.8	117.5	7.20, d, 1.7	117.3
**3′**	C	-	146.8^e^	-	146.0 ^h^
**4′**	C	-	145.8^f^	-	144.1
**5′**	CH	6.96, d, 8.4	118.4	7.0, d, 8.8	117.8
**6′**	CH	7.12, dd, 8.4, 1.8	123.1	7.19, dd, 8.8, 1.7	123.6
**7′**	CH	7.57, d, 16.0	146.3	7.57, d, 16.0	146.0 ^h^
**8′**	CH	6.38, d, 16.0	117.2	6.39, d, 16.0	116.0
**9′**	C	-	168.8	-	169.2
**1″**	C	-	131.3	-	131.0
**2″**	CH	6.80^c^	117.1	6.85^d^	119.6
**3″**	C	-	145.8 ^f^	-	144.9
**4″**	C	-	145.1	-	144.0 ^g^
**5″**	CH	6.70, d, 8.1	116.3	6.80, d, 8.1	116.4
**6″**	CH	6.66, dd, 8.1, 1.8	121.7	6.74, dd, 8.1, 1.8	121.7
**7″**	CH_2_	2.95, dd, 14.3, 10.0	38.9	2.96, dd, 14.3, 10.0	37.9
		3.12, dd, 14.3, 3.2		3.13, dd, 14.3, 3.4	
**8″**	CH	5.11, dd, 10.0, 3.2	77.9	5.04, dd, 10.0, 3.4	77.3
**9″**	C	-	176.9	-	177.5

^a^ CD_3_OD, ^b^ CD_3_OD:D_2_O 1:1, ^c–h^ overlapped signal.

**Table 3:** Validation parameters for the quantitative UHPLC MS method on polyphenolic standards.

Standard Compound	t_R_	[M − H]^−^ *m/z*	Error (ppm)	MS/MS *m/z*	R^2^	Working Range (µg/mL)	LOD (ng/mL)	LOQ (ng/mL)	QC (µg/mL)	Precision (% RSD)		Accuracy (%)	
										Intra-Day	Inter-Day	Intra-Day	Inter-Day
									0.250	10.8	9.1	98.4	96.7
**Caffeic acid**	1.03	179.03443	1.90	135.04	0.9976	0.250–10	43.7	132.6	1	7.6	6.4	100.7	109.8
									10	4.9	6.5	94.1	106.4
									0.125	7.8	7.7	94.0	96.2
**Luteolin-7-rutinoside**	1.56	593.15064	2.26	285.04	0.9977	0.125–10	29.8	90.4	1	5.4	6.4	100.0	101.8
									10	2.9	4.4	100.9	99.5
									0.125	2.8	4.7	96.3	99.4
**Luteolin-7-glucoside**	1.64	447.09273	1.81	285.04	0.9960	0.125–5	17.0	35.5	1	2.4	2.4	115.0	116.0
									5	2.5	2.7	105.5	106.6
**Luteolin-7-glucuronide**									0.125	10.8	9.6	96.3	94.8
	1.67	461.07200	1.84	285.04	0.9983	0.125–5	39.8	120.7	1	7.9	8.0	98.8	107.6
									5	4.4	7.0	94.1	108.4
									0.125	3.9	5.8	90.3	94.0
**Naringin**	1.85	579.17138	1.48	151.00	0.9981	0.125–10	6.1	18.3	1	4.3	4.1	99.7	101.4
									10	6.2	6.9	97.8	101.3
									0.125	5.6	7.0	85.5	89.5
**Hesperidin**	1.95	609.18194	0.61	301.07	0.9973	0.125–5	3.3	10.1	1	4.4	4.6	104.7	107.0
									5	3.9	6.1	101.3	104.5
									0.125	2.0	3.3	87.4	88.6
**Apigenin-7-glucoside**	1.99	431.09782	0.74	268.04	0.9984	0.125–5	25.1	76.2	1	2.4	2.1	108.6	109.1
									5	2.6	3.3	107.8	109.5
									0.125	3.2	5.9	90.1	94.0
**Diosmin**	2.01	607.16629	1.35	299.06	0.9993	0.125–5	4.7	14.4	1	4.7	4.8	106.9	109.4
									5	3.1	4.5	104.1	107.0
									0.125	1.2	5.2	89.1	92.7
**Rosmarinic acid**	2.08	359.07669	1.86	161.02	0.9978	0.125–10	6.3	19.0	1	2.7	2.6	104.3	105.6
									10	3.9	4.2	101.7	103.4
									0.250	6.3	10.3	100.3	99.8
**Salvianolic acid B**	2.49	717.14555	2.24	321.04	0.9989	0.250–10	75.5	229.0	1	12.7	14.3	93.4	93.5
									10	12.2	14.9	100.8	101.9
									0.125	9.4	13.8	94.0	95.3
**Salvianolic acid A**	2.86	493.11347	2.33	185.02	0.9977	0.125–10	11.3	34.2	1	11.8	14.1	86.9	89.8
									10	7.3	14.7	103.4	105.8
									0.250	12.2	11.0	100.1	95.2
**Luteolin**	3.03	285.03991	1.96	133.03	0.9966	0.250–5	47.0	142.6	1	9.6	7.5	105.1	113.9
									5	5.9	5.9	94.4	106.6

**Table 4:** Absolute quantitative composition (mg/g extract) of polyphenols in ten *M. rotundifolia* populations from different geographical areas. Values are means of three replicates ± SD.

Peak	Compound	t_R_	[M − H]^−^ *m/z*	MS/MS *m/z*	MROT-1	MROT-2	MROT-3	MROT-4	MROT-5	MROT-6	MROT-7	MROT-8	MROT-9	MROT-10
**1**	Caffeic acid	1.05	179.03443	135.04	1.21 ± 0.01	1.16 ± 0.10	0.13 ± 0.05	0.60 ± 0.09	0.96 ± 0.14	0.94 ± 0.01	1.38 ± 0.16	0.85 ± 0.14	0.70 ± 0.12	0.38 ± 0.02
**2**	Luteolin-7-rutinoside	1.56	593.15064	285.04	2.67 ± 0.68	6.87 ± 0.69	-	0.79 ± 0.10	1.84 ± 0.15	1.86 ± 0.21	8.02 ± 0.47	2.05 ± 0.03	1.62 ± 0.26	1.36 ± 0.02
**3**	Luteolin-7-glucoside	1.64	447.09273	285.04	3.84 ± 0.49	4.60 ± 0.24	-	0.53 ± 0.06	2.39 ± 0.30	1.62 ± 0.02	3.66 ± 0.78	1.23 ± 0.20	0.64 ± 0.03	0.57 ± 0.01
**4**	Luteolin-7-glucuronide	1.67	461.07200	285.04	26.34 ± 7.88	22.71 ± 2.10	1.91 ± 0.63	5.16 ± 0.94	8.73 ± 0.40	15.26 ± 1.91	9.71 ± 3.49	4.39 ± 0.74	1.75 ± 0.17	1.81 ± 0.16
**5**	Isonaringin	1.77	579.17138	271.06	0.25 ± 0.03	-	-	0.48 ± 0.14	-	-	0.67 ± 0.02	-	-	-
**6**	Hesperidin	1.95	609.18194	301.07	2.05 ± 0.15	-	9.54 ± 2.29	10.00 ± 3.46	3.58 ± 0.93	-	-	-	-	-
**7**	Apigenin-7-glucoside	1.98	431.09782	268.04	0.70 ± 0.03	0.67 ± 0.03	-	-	0.23 ± 0.02	0.21 ± 0.01	-	-	-	-
**8**	Salvianolic acid L	2.00	717.14555	295.06	276.44 ± 91.65	103.72 ± 2.17	2.02 ± 0.89	23.70 ± 4.69	66.31 ± 4.06	74.05 ± 8.68	29.92 ± 5.02	14.82 ± 3.44	6.27 ± 1.03	10.86 ± 0.51
**9**	Diosmin	2.02	607.16629	299.06	-	1.70 ± 0.68	3.95 ± 0.37	-	-	-	-	-	-	-
**10**	Rosmarinic acid	2.08	359.07669	161.02	116.15 ± 6.57	65.65 ± 0.39	6.53 ± 1.46	29.00 ± 0.99	68.99 ± 11.16	87.89 ± 8.52	54.51 ± 9.72	37.68 ± 4.82	50.21 ± 6.35	23.97 ± 1.15
**11**	Luteolin-3’-glucuronide	2.25	461.07200	285.04	42.59 ± 15.06	37.43 ± 1.05	3.50 ± 0.34	8.40 ± 1.32	15.62 ± 0.82	32.44 ± 3.36	16.59 ± 5.55	7.61 ± 1.28	3.61 ± 0.31	4.31 ± 0.28
**12**	Isosalvianolic acid B	2.30	717.14555	357.06	61.10 ± 24.35	-	-	-	-	21.75 ± 0.70	-	-	-	2.35 ± 0.19
**13**	Isosalvianolic acid W	2.44	537.10385	135.04	28.23 ± 8.89	3.24 ± 0.01	-	2.27 ± 0.53	9.95 ± 0.57	7.80 ± 0.22	5.19 ± 2.25	2.68 ± 0.47	3.98 ± 0.37	3.37 ± 0.0.01
**14**	Salvianolic acid B	2.52	717.14555	321.04	-	10.07 ± 2.97	1.32 ± 0.37	2.06 ± 0.08	5.22 ± 0.92	-	0.27 ± 0.01	0.50 ± 0.04	1.55 ± 0.01	-
**15**	Salvianolic acid W	2.95	537.10385	161.02	71.34 ± 15.14	10.28 ± 0.97	2.05 ± 0.43	8.83 ± 0.66	25.91 ± 0.38	21.89 ± 1.04	5.99 ± 1.99	1.92 ± 0.22	2.57 ± 0.24	2.62 ± 0.27
**16**	Luteolin	3.05	285.03991	133.03	2.25 ± 0.03	4.78 ± 0.44	4.91 ± 0.19	4.01 ± 0.33	3.01 ± 0.60	0.76 ± 0.25	3.68 ± 0.27	1.53 ± 0.12	0.10 ± 0.02	0.36 ± 0.04
**17**	Isosalvianolic acid A	3.17	493.11347	135.04	18.27 ± 3.30	2.96 ± 0.11	1.24 ± 0.17	2.98 ± 0.09	7.12 ± 0.97	3.74 ± 0.01	2.78 ± 0.90	1.31 ± 0.17	1.83 ± 0.48	0.92 ± 0.04

**Table 5:** Correlation coefficients between identified compounds and biological activities.

	DPPH	β-carotene	FRAP	AChE
Polyphenols	−0.70 **	−0.36 *	0.45 *	−0.21 ^ns^
Flavonoids	−0.73 **	0.14 ^ns^	0.34 *	−0.43 *
**Phenolic acids**				
Caffeic acid	−0.48 *	−0.32 *	0.14 ^ns^	−0.22 ^ns^
Salvianolic acid L	−0.56 **	−0.49 *	0.30 *	−0.15 ^ns^
Rosmarinic acid	−0.47 *	−0.49 *	0.07 ^ns^	−0.16 ^ns^
Isosalvianolic acid B	−0.41 *	−0.51 **	0.31 *	0.06 ^ns^
Isosalvianolic acid W	−0.51 **	−0.30 *	0,41 *	−0.03 ^ns^
Salvianolic acid B	−0.12 ^ns^	−0.03 ^ns^	−0.30 *	−0.49 **
Salvianolic acid W	−0.58 **	−0.31 *	0.34 *	−0.16 ^ns^
Isosalvianolic acid A	−0.61 **	−0.21 ^ns^	0.41 *	−0.19 ^ns^
**Flavonoids**				
Luteolin	−0.37 *	0.29 ^ns^	−0.12 ^ns^	−0.65 **
Luteolin-7-rutinoside	−0.25 ^ns^	−0.26 ^ns^	0.002 ^ns^	−0.07 ^ns^
Luteolin-7-glucoside	−0.45 *	−0.39 *	0.07 ^ns^	−0.23 ^ns^
Luteolin-7-glucuronide	−0.51 **	−0.61 **	0.05 ^ns^	−0.24 ^ns^
Luteolin-3’-glucuronide	−0.45 *	−0.65 **	−0.02 ^ns^	−0.20 ^ns^
Apigenin-7-glucoside	−0.40 *	−0.58 **	0.03 ^ns^	−0.23 ^ns^
Diosmin	0.26 ^ns^	0.11 ^ns^	−0.54 **	−0.36 *
Isonaringin	−0.65 **	0.30 *	0.53 **	−0.09 ^ns^
Hesperidin	−0.25 ^ns^	0.56 **	−0.004 ^ns^	−0.48 *

*, ** = significant at *p* < 0.05 and *p* < 0.01, respectively; ns = not significant.

## Supplementary Materials

The following are available online at https://www.mdpi.com/1420-3049/24/13/2351/s1, Table S1. Geographical distribution of the investigated populations of *M. rotundifolia*. Figure S1. ^1^H NMR spectrum (600 MHz, CD_3_OD) of luteolin-3′-glucuronide. Figure S2. ^1^H,^1^H- COSY NMR spectrum (600 MHz, CD_3_OD) of luteolin-3′-glucuronide. Figure S3. ^1^H,^1^H TOCSY- NMR spectrum (600 MHz, CD_3_OD) of luteolin-3′-glucuronide. Figure S4. HSQC-edited NMR spectrum (600 MHz, CD_3_OD) of luteolin-3′-glucuronide. Figure S5. HMBC NMR spectrum (600 MHz, CD_3_OD) of luteolin-3′-glucuronide. Figure S6. ^1^H NMR spectrum (400 MHz, CD_3_OD) of salvianolic acid L. Figure S7. ^1^H,^1^H- COSY NMR spectrum (400 MHz, CD_3_OD) of salvianolic acid L. Figure S8. HSQC-edited NMR spectrum (400 MHz, CD_3_OD) of salvianolic acid L. Figure S9. HMBC NMR spectrum (400 MHz, CD_3_OD) of salvianolic acid L. Figure S10. ^1^H NMR spectrum (400 MHz, CD_3_OD/D_2_O) of salvianolic acid L. Figure S11. ^1^H NMR spectrum (600 MHz, CD_3_OD) of salvianolic acid W. Figure S12. ^1^H,^1^H- COSY NMR spectrum (400 MHz, CD_3_OD) of salvianolic acid W. Figure S13. HSQC-edited NMR spectrum (600 MHz, CD_3_OD) of salvianolic acid W. Figure S14. HMBC NMR spectrum (600 MHz, CD_3_OD) of salvianolic acid W. Figure S15. ^13^C NMR spectrum (100 MHz, CD_3_OD) of salvianolic acid W. Figure S16. ^1^H NMR spectrum (400 MHz, CD_3_OD/D_2_O) of salvianolic acid W. Figure S17. ^1^H,^1^H- COSY NMR spectrum (600 MHz, CD_3_OD/D_2_O) of salvianolic acid W Figure S18. HSQC-edited NMR spectrum (600 MHz, CD_3_OD/D_2_O) of salvianolic acid W. Figure S19. HMBC NMR spectrum (600 MHz, CD_3_OD/D_2_O) of salvianolic acid W. Figure S20. CD spectrum of salvianolic acid W. Figure S21. Calibration curve for Total Phenolic Content (TPC) analysis. Figure S22. Calibration curve for Total Flavonoid Content (TFC) analysis.
