# Supplementary material for: Polyphenolic Profiling, Quantitative Assessment and Biological Activities of Tunisian Native Mentha rotundifolia (L.) Huds
Source: Molecules. 2019 Jun 26;24(13):2351. doi: 10.3390/molecules24132351 (PMC6650961; doi:10.3390/molecules24132351)

SUPPORTING INFORMATION

Polyphenolic Profiling, Quantitative Assessment and Biological Activities of Tunisian Native *Mentha rotundifolia* (L.) Huds.

Imen Ben Haj Yahia [1,†], Yosr Zaouali [1,†], Maria Letizia Ciavatta [2], Alessia Ligresti [2], Rym Jaouadi [1], Mohamed Boussaid [1] and Adele Cutignano [2,*]

[1] Department of Biology, National Institute of Applied Science and Technology, B.P. 676, 1080, Tunis Cedex, Tunisia; imenbenhajyahia@gmail.com (I.B.H.Y.); zaoualiyosrinsat@gmail.com (Y.Z.); jaouadi.ryma@gmail.com (R.J.); mohamed.boussaid@insat.rnu.tn (M.B.)

[2] Institute of Biomolecular Chemistry (ICB), National Research Council (CNR), 80078 Pozzuoli (NA), Italy; lciavatta@icb.cnr.it (M.L.C.); aligresti@icb.cnr.it (A.L.)

* Correspondence: adele.cutignano@icb.cnr.it; Tel.: +39-081-8675313; Fax: +39-081-8041770

† These authors contributed equally to this work.

Table S1. Geographical distribution of the investigated populations of *M. rotundifolia*.

Figure S1. ^{1}H NMR spectrum (600 MHz, CD_3OD) of luteolin-3’-glucuronide

Figure S2. ^{1}H,^{1}H- COSY NMR spectrum (600 MHz, CD_3OD) of luteolin-3’-glucuronide

Figure S3. ^{1}H,^{1}H TOCSY- NMR spectrum (600 MHz, CD_3OD) of luteolin-3’-glucuronide

Figure S4. HSQC-edited NMR spectrum (600 MHz, CD_3OD) of luteolin-3’-glucuronide

Figure S5. HMBC NMR spectrum (600 MHz, CD_3OD) of luteolin-3’-glucuronide

Figure S6. ^{1}H NMR spectrum (400 MHz, CD_3OD) of salvianolic acid L

Figure S7. ^{1}H,^{1}H- COSY NMR spectrum (400 MHz, CD_3OD) of salvianolic acid L

Figure S8. HSQC-edited NMR spectrum (400 MHz, CD_3OD) of salvianolic acid L

Figure S9. HMBC NMR spectrum (400 MHz, CD_3OD) of salvianolic acid L

Figure S10. ^{1}H NMR spectrum (400 MHz, CD_3OD/D_2O) of salvianolic acid L

Figure S11. ^{1}H NMR spectrum (600 MHz, CD_3OD) of salvianolic acid W

Figure S12. ^{1}H,^{1}H- COSY NMR spectrum (400 MHz, CD_3OD) of salvianolic acid W

Figure S13. HSQC-edited NMR spectrum (600 MHz, CD_3OD) of salvianolic acid W

Figure S14. HMBC NMR spectrum (600 MHz, CD_3OD) of salvianolic acid W

Figure S15. ^{13}C NMR spectrum (100 MHz, CD_3OD) of salvianolic acid W

Figure S16. ^{1}H NMR spectrum (400 MHz, CD_3OD/D_2O) of salvianolic acid W

Figure S17. ^{1}H,^{1}H- COSY NMR spectrum (600 MHz, CD_3OD/D_2O) of salvianolic acid W

Figure S18. HSQC-edited NMR spectrum (600 MHz, CD_3OD/D_2O) of salvianolic acid W

Figure S19. HMBC NMR spectrum (600 MHz, CD_3OD/D_2O) of salvianolic acid W

Figure S20. CD spectrum of salvianolic acid W

Figure S21. Calibration curve for Total Phenolic Content (TPC) analysis

Figure S22. Calibration curve for Total Flavonoid Content (TFC) analysis

Table S1. Geographical distribution of the investigated populations of *M. rotundifolia*. Lh: lower humid; Usa: upper semi-arid.

Code	Gouvernorate	Locality	Bioclimatic zone	Latitude (N)	Longitude (E)	Altitude (m)
MROT-1	Beja	Tamra	Lh	37°10'	9°08'	202
MROT-2	Beja	Oued El Maaden	Lh	36°47'	8°27'	220
MROT-3	Beja	Goussa	Lh	36°78'	9°08'	504
MROT-4	Bizerta	SidiNsir	Lh	36°92'	9°38'	285
MROT-5	Beja	Wechteta	Lh	36°48'	8°21'	200
MROT-6	Beja	OuedZarga	Usa	36°4'	9°26'	109
MROT-7	Siliana	Kesra	Usa	35°48'	9°21'	850
MROT-8	Siliana	Aïn Cristal	Usa	36°61'	9°20'	428
MROT-9	Nabeul	MenzelBouzalfa	Usa	36°41'	10°35'	637
MROT-10	Beja	Teboursek	Usa	36°27'	9°14'	436

Figure S1. ^{1}H NMR spectrum (600 MHz, CD_3OD) of luteolin-3'-glucuronide

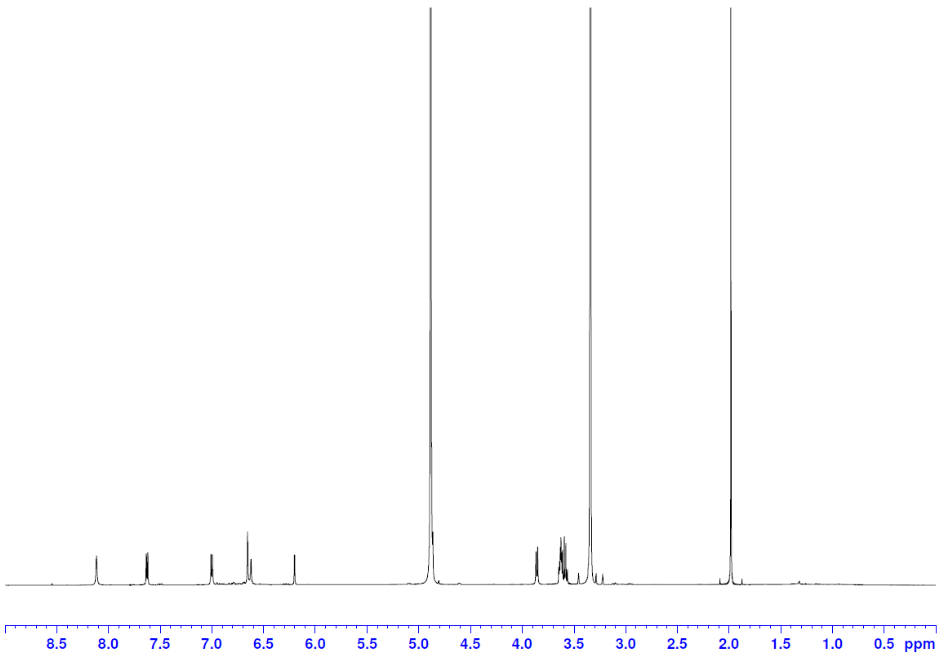

Figure S2. ^{1}H,^{1}H- COSY NMR spectrum (600 MHz, CD_3OD) of luteolin-3'-glucuronide

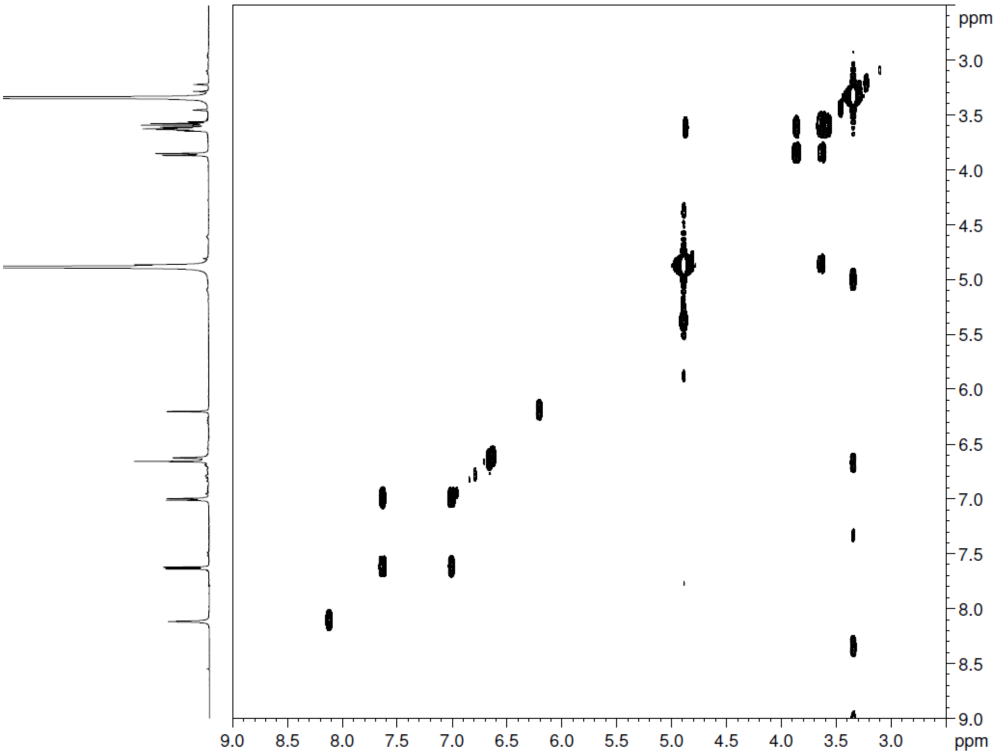

Figure S3. ^{1}H,^{1}H TOCSY- NMR spectrum (600 MHz, CD_3OD) of luteolin-3'-glucuronide

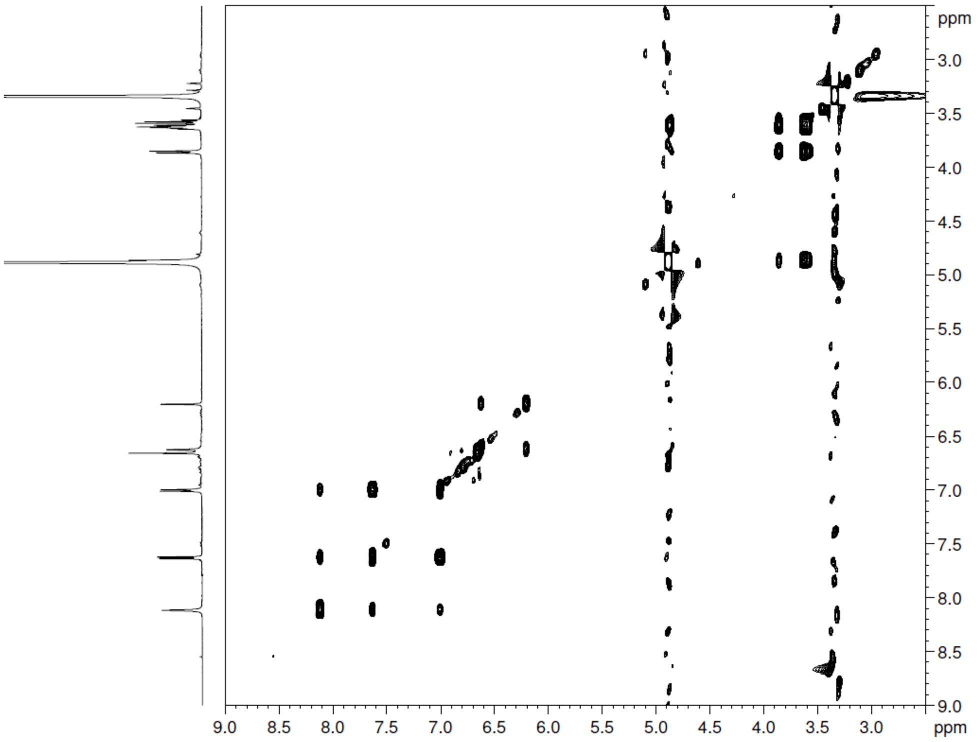

Figure S4. HSQC-edited NMR spectrum (600 MHz, CD_3OD) of luteolin-3'-glucuronide

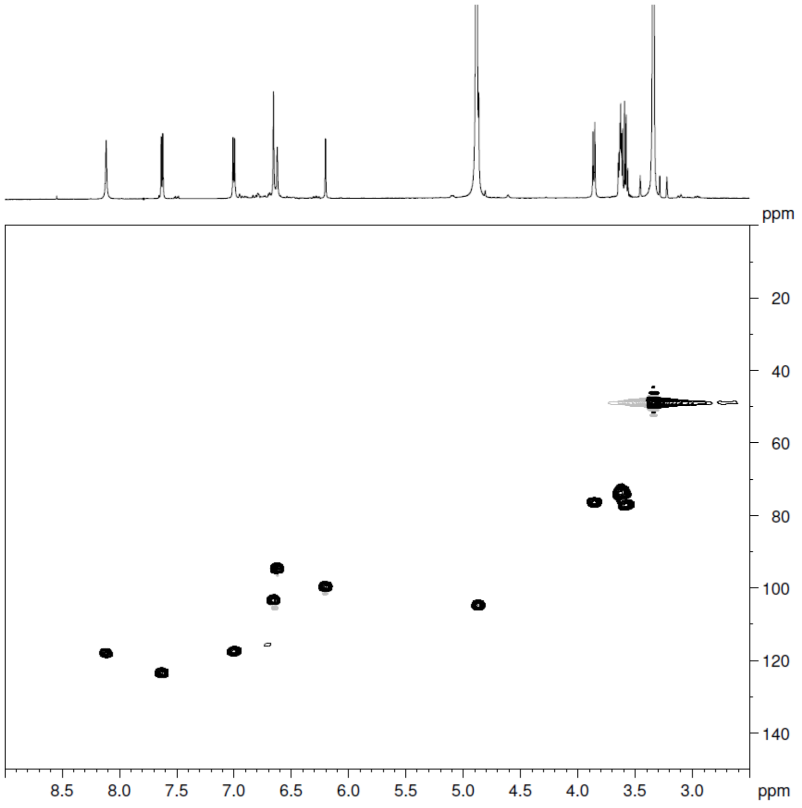

Figure S5. HMBC NMR spectrum (600 MHz, CD_3OD) of luteolin-3'-glucuronide

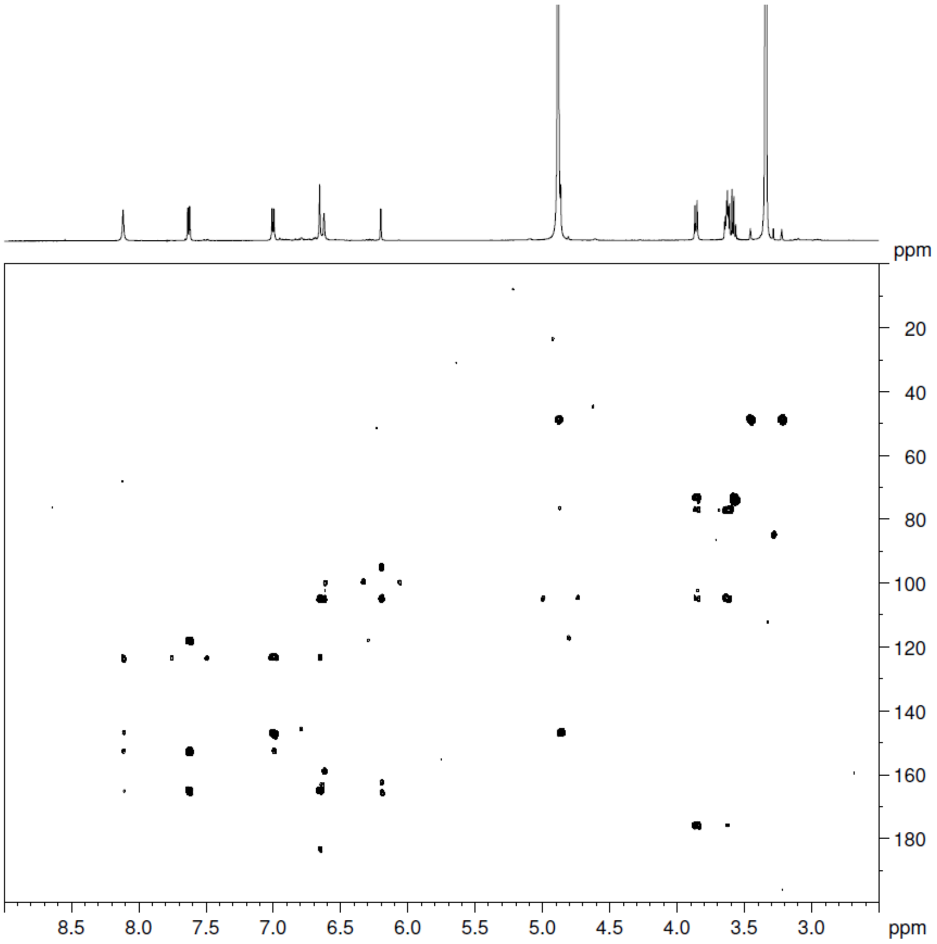

Figure S6. ^{1}H NMR spectrum (400 MHz, CD_3OD) of salvianolic acid L

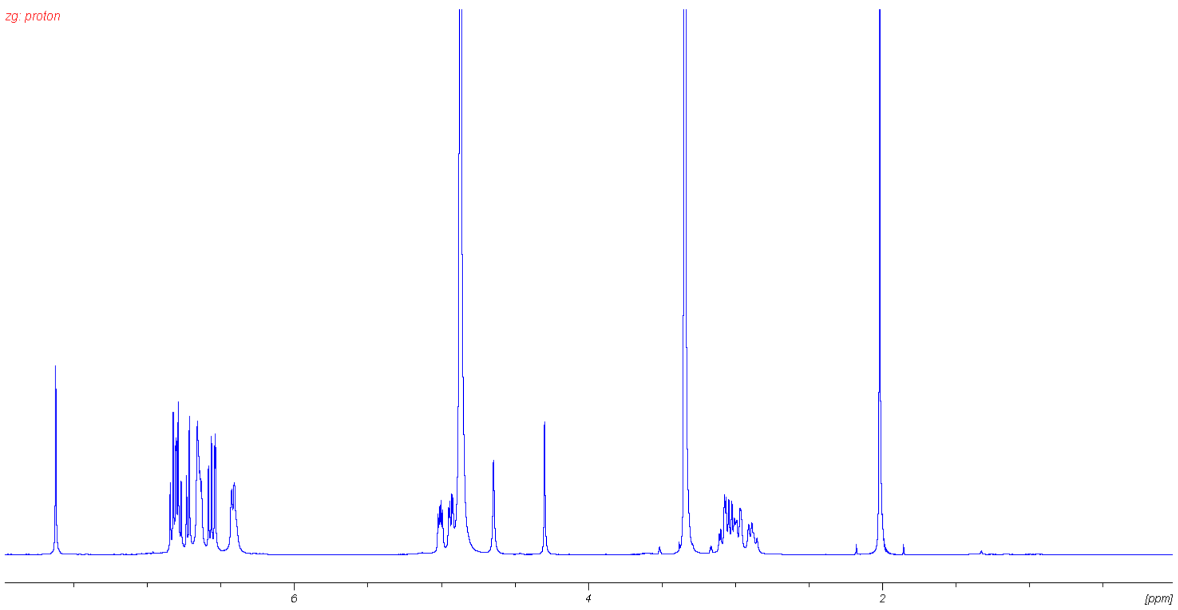

Figure S7. ^{1}H,^{1}H- COSY NMR spectrum (400 MHz, CD_3OD) of salvianolic acid L

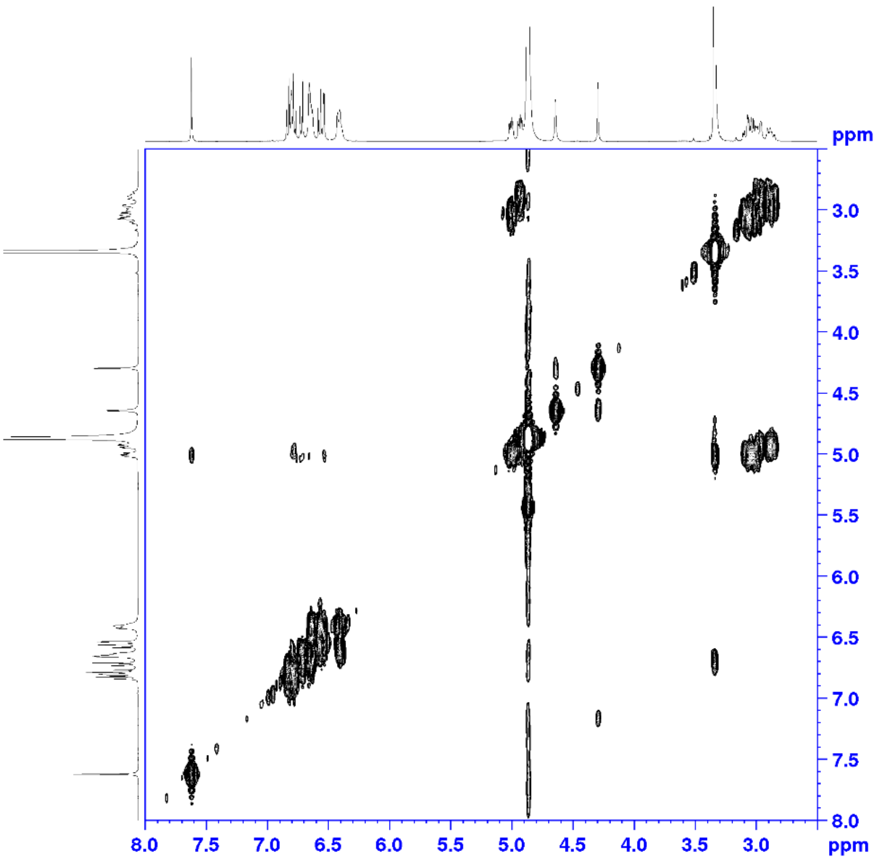

Figure S8. HSQC-edited NMR spectrum (400 MHz, CD_3OD) of salvianolic acid L

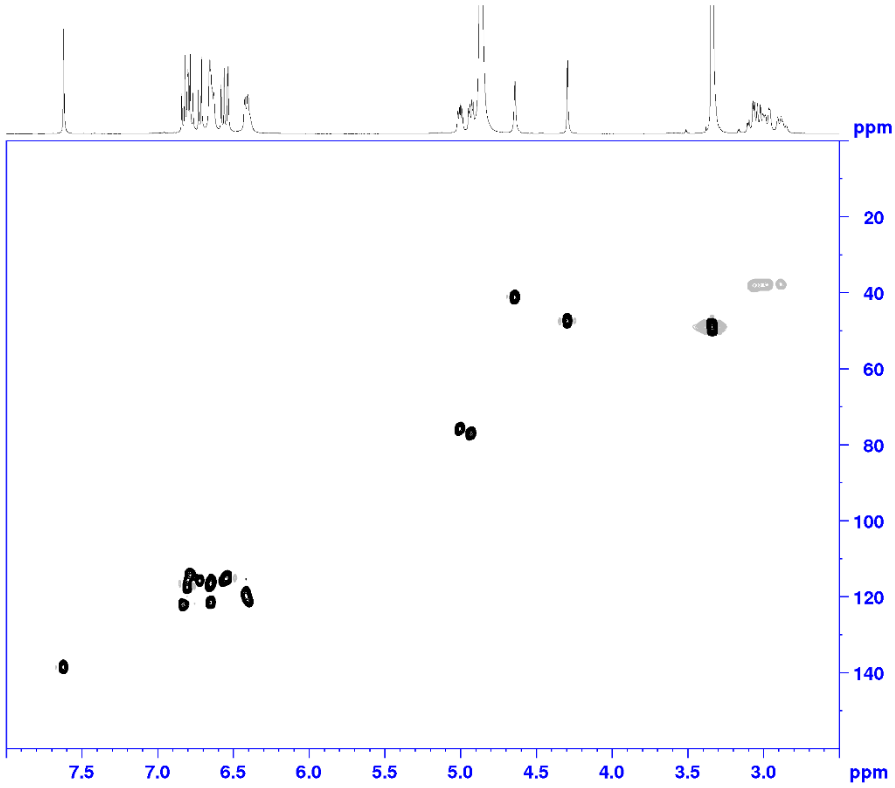

Figure S9. HMBC NMR spectrum (400 MHz, CD_3OD) of salvianolic acid L

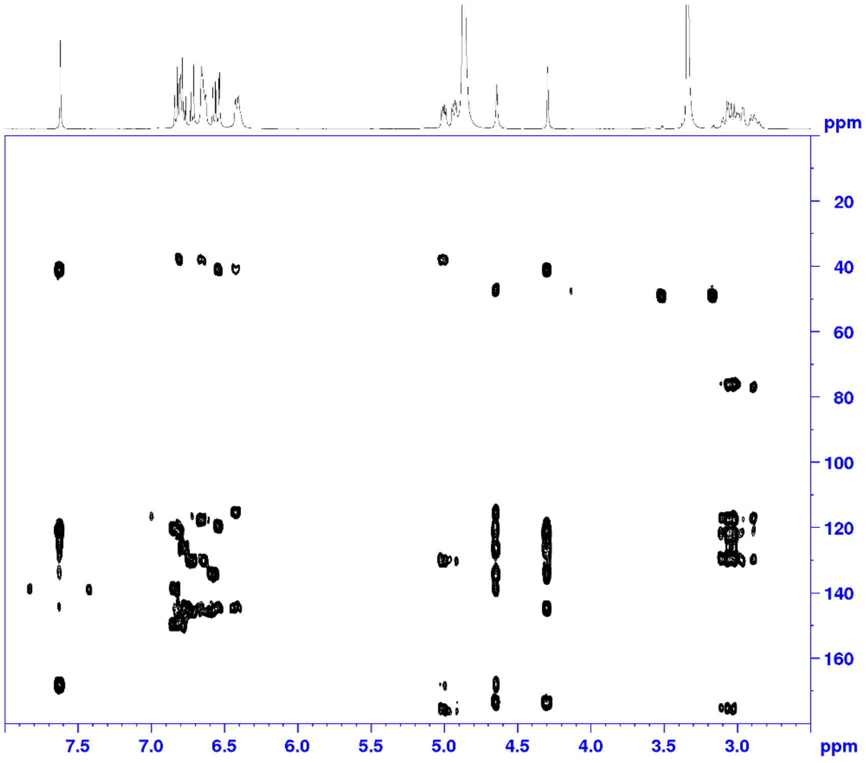

Figure S10. ^{1}H NMR spectrum (400 MHz, CD_3OD/D_2O) of salvianolic acid L

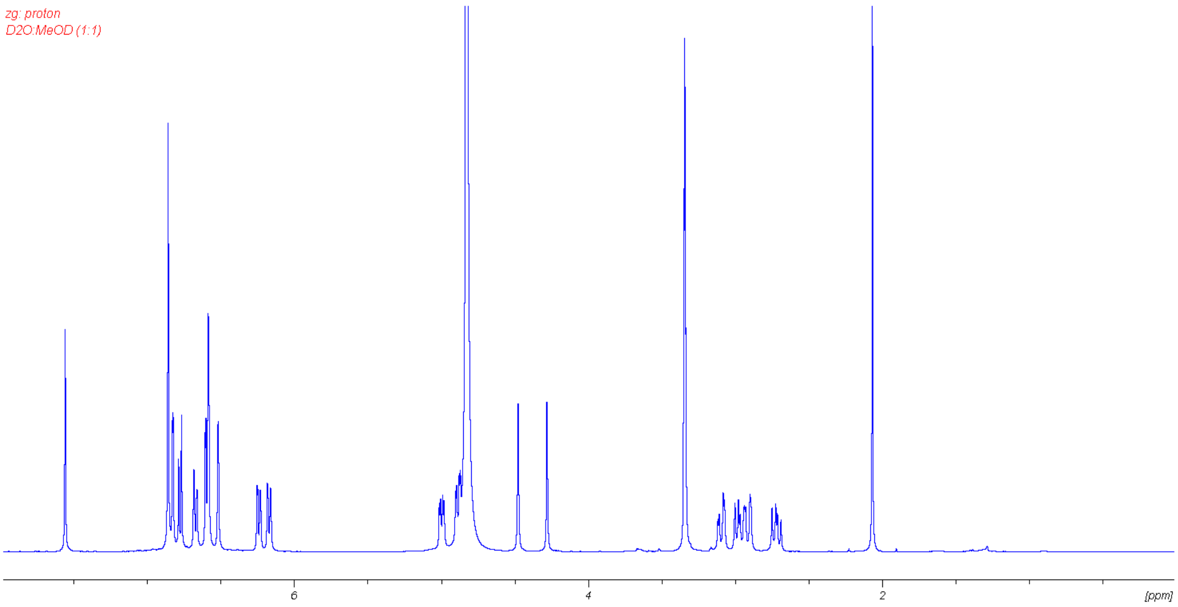

Figure S11. ^{1}H NMR spectrum (600 MHz, CD_3OD) of salvianolic acid W

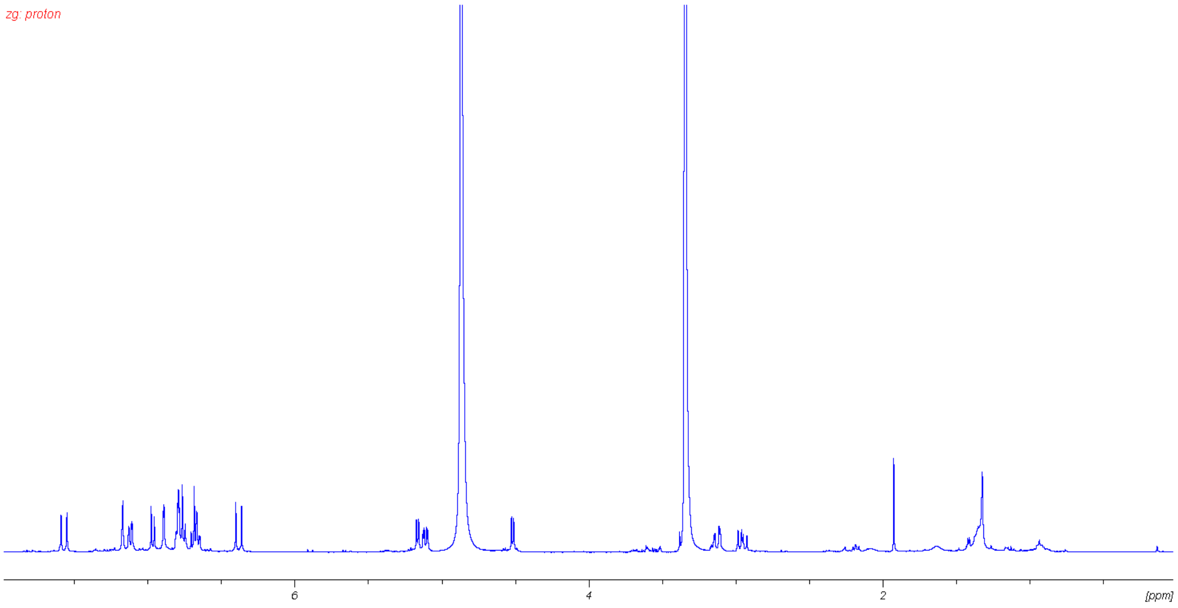

Figure S12. $^{1}H,^{1}H$- COSY NMR spectrum (400 MHz, CD_3OD) of salvianolic acid W

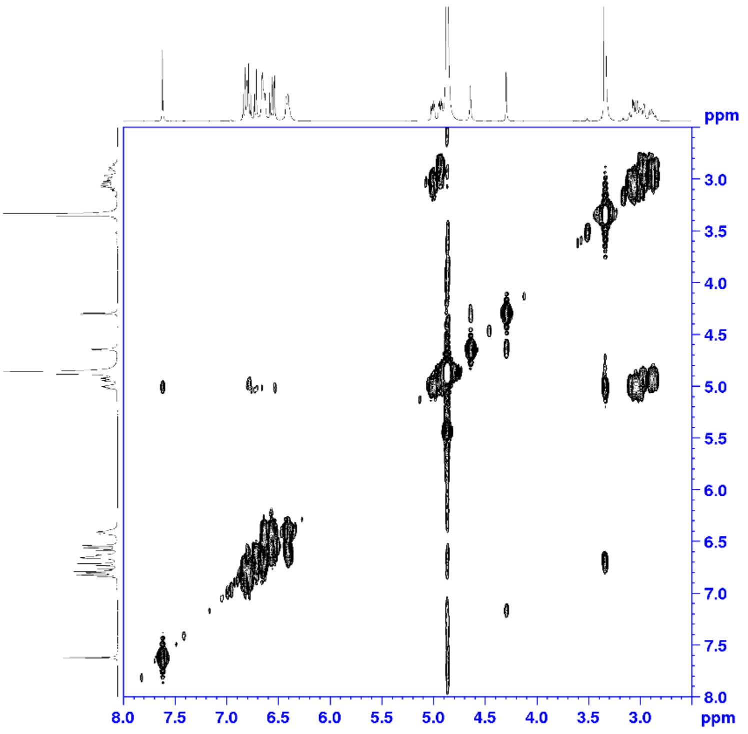

Figure S13. HSQC-edited NMR spectrum (600 MHz, CD_3OD) of salvianolic acid W

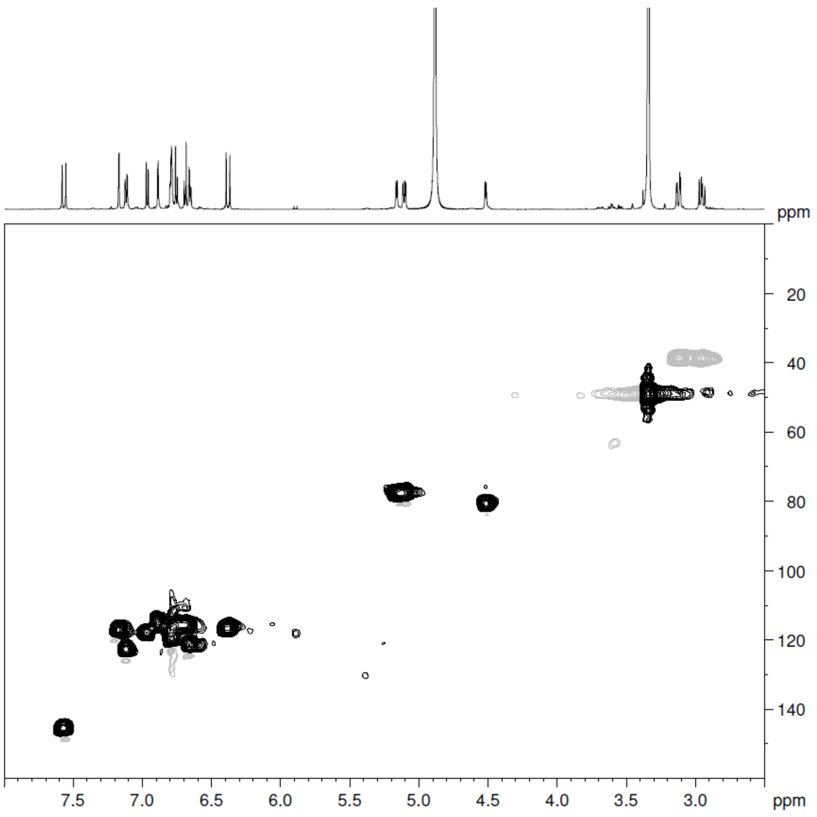

Figure S14. HMBC NMR spectrum (600 MHz, CD_3OD) of salvianolic acid W

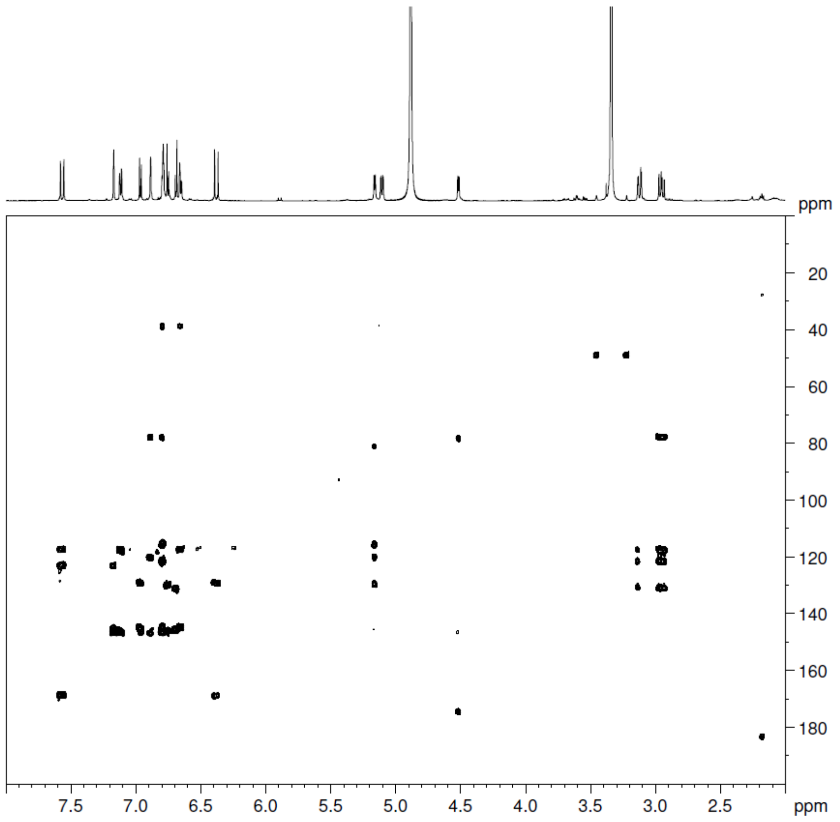

Figure S15. ^{13}C NMR spectrum (100 MHz, CD_3OD) of salvianolic acid W

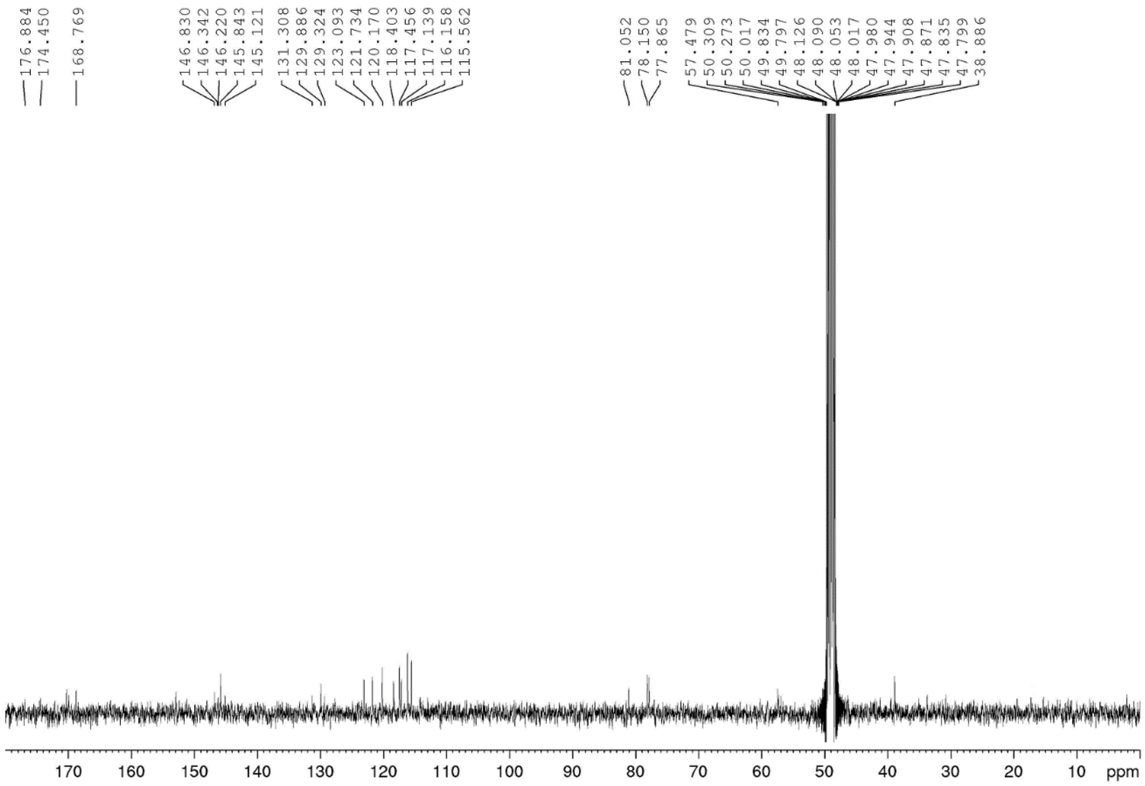

Figure S16. ^{1}H NMR spectrum (400 MHz, CD_3OD/D_2O) of salvianolic acid W

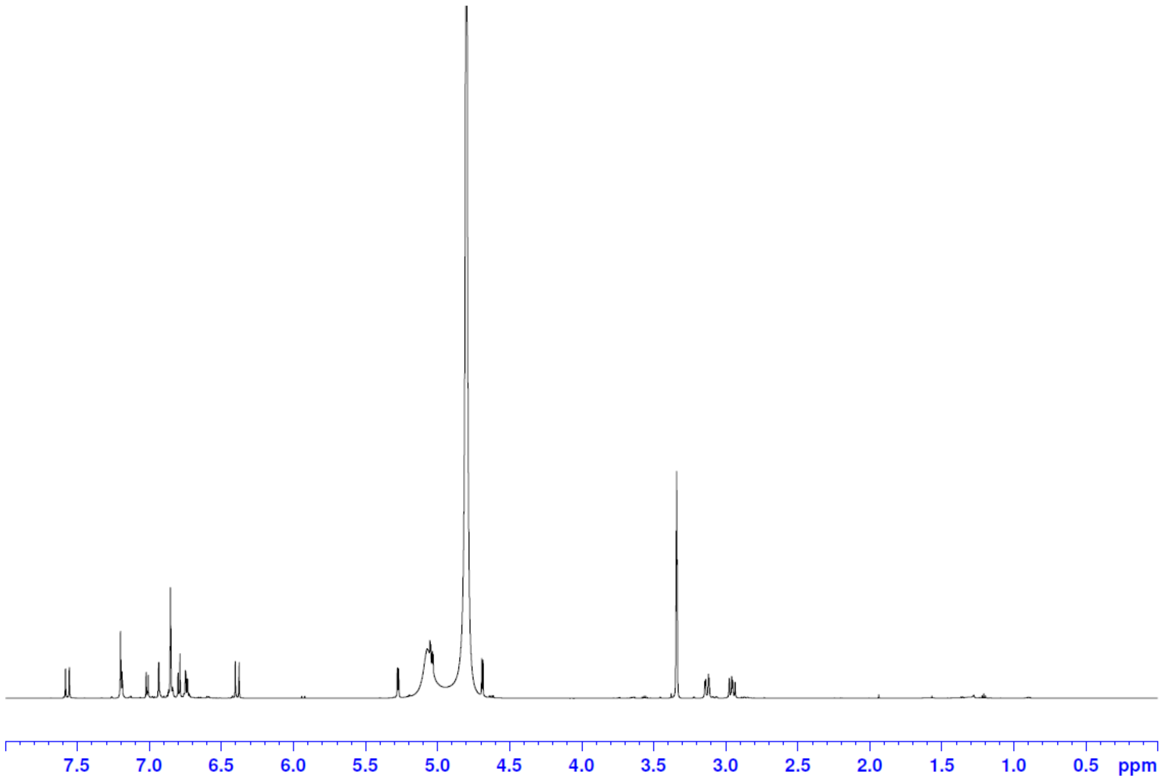

Figure S17. $^{1}H,^{1}H$- COSY NMR spectrum (600 MHz, CD_3OD/D_2O) of salvianolic acid W

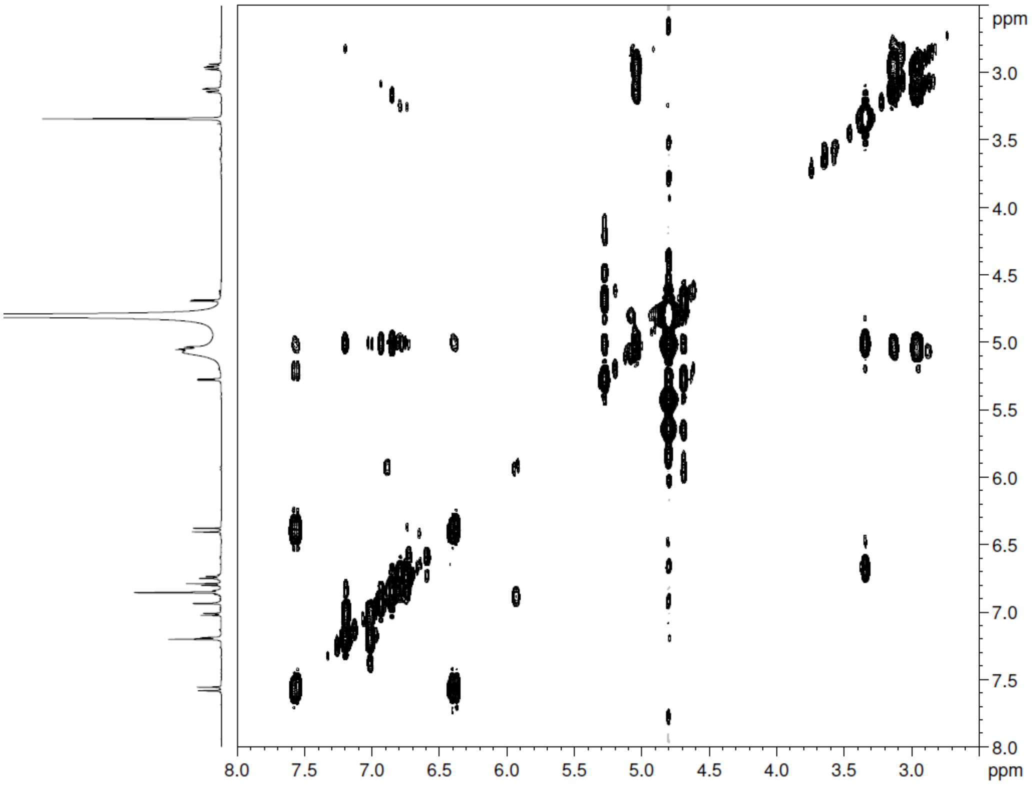

Figure S18. HSQC-edited NMR spectrum (600 MHz, CD_3OD/D_2O) of salvianolic acid W

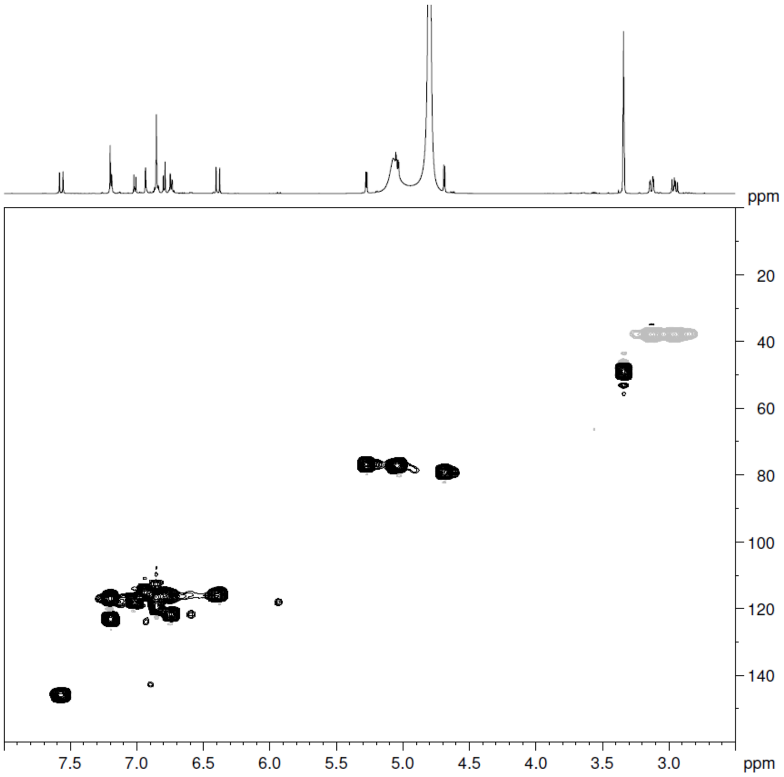

Figure S19. HMBC NMR spectrum (600 MHz, CD_3OD/D_2O) of salvianolic acid W

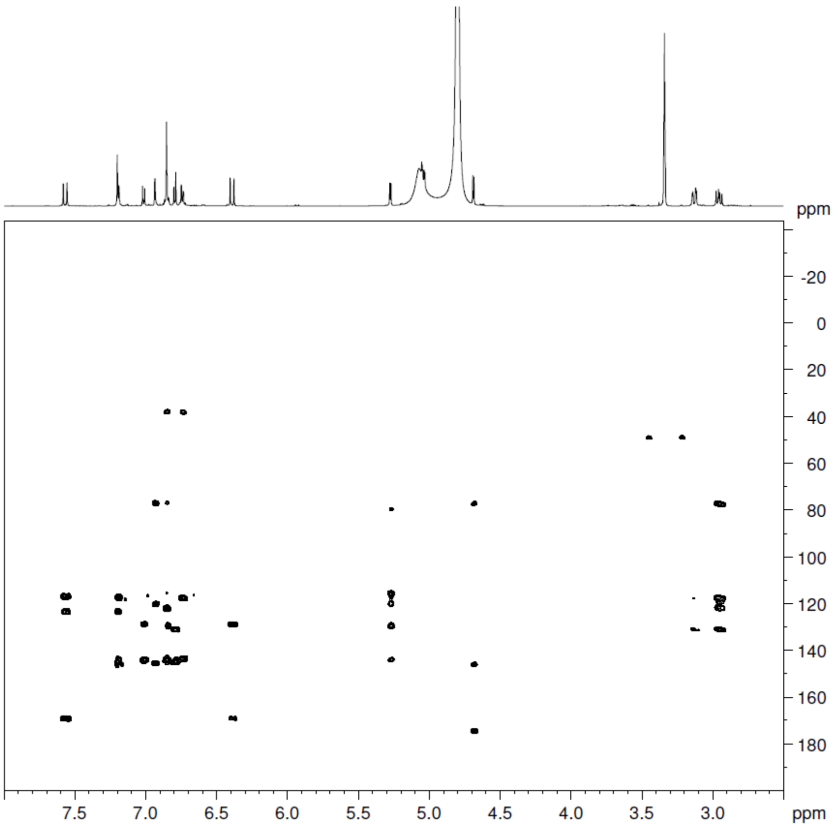

Figure S20. CD spectrum of salvianolic acid W

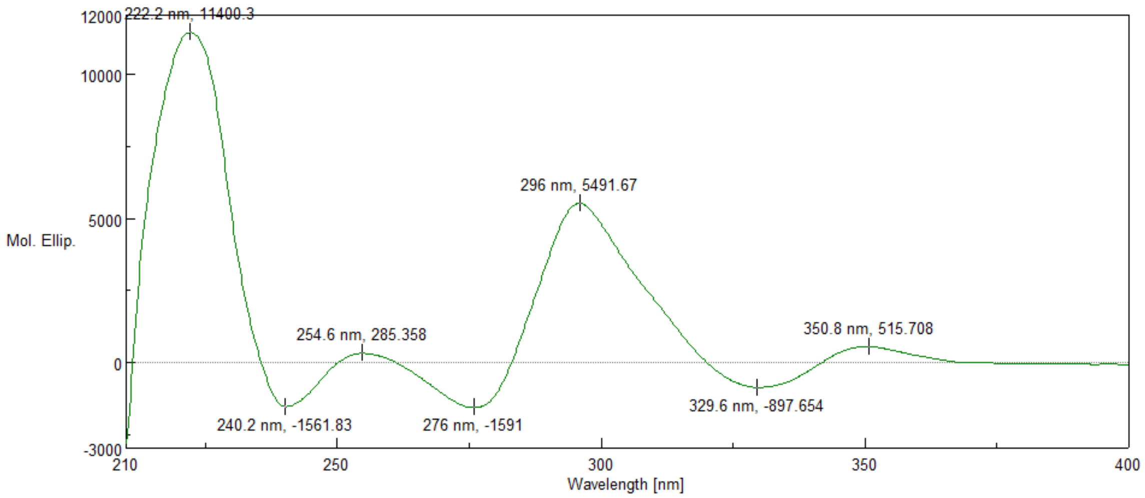

Figure S21. Calibration curve for Total Phenolic Content (TPC) analysis

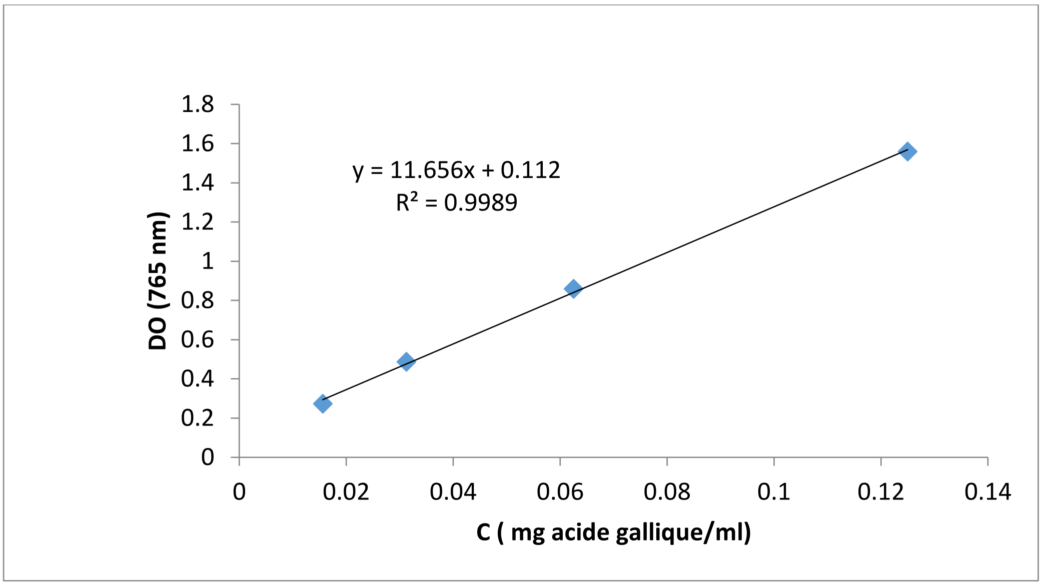

Figure S22. Calibration curve for Total Flavonoid Content (TFC) analysis

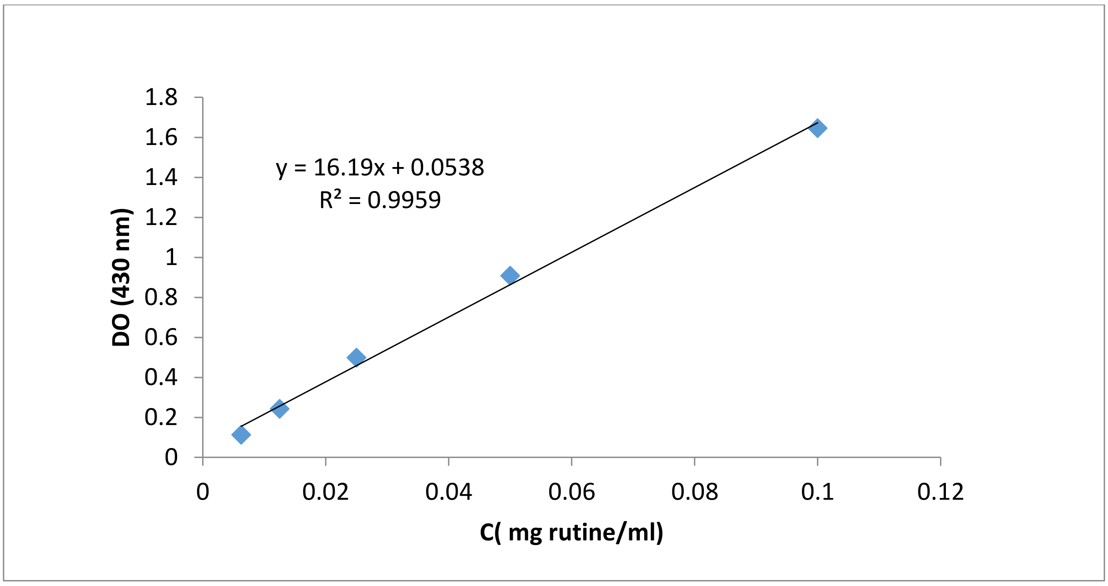

Supplement: Supplementary file 1 [file molecules-24-02351-s001.pdf]
